# A systematic review and meta-analysis of CT and MRI radiomics in ovarian cancer: methodological issues and clinical utility

**DOI:** 10.1186/s13244-023-01464-z

**Published:** 2023-07-03

**Authors:** Meng-Lin Huang, Jing Ren, Zheng-Yu Jin, Xin-Yu Liu, Yong-Lan He, Yuan Li, Hua-Dan Xue

**Affiliations:** 1 Department of Radiology, Peking Union Medical College Hospital, Chinese Academy of Medical Sciences & Peking Union Medical College, Beijing, People’s Republic of China; 2 Department of Obstetrics and Gynecology, Peking Union Medical College Hospital, Chinese Academy of Medical Sciences & Peking Union Medical College, National Clinical Research Center for Obstetric & Gynecologic Diseases, Beijing, People’s Republic of China

**Keywords:** Ovarian neoplasms, Differential diagnosis, Machine learning, Systematic review

## Abstract

**Objectives:**

We aimed to present the state of the art of CT- and MRI-based radiomics in the context of ovarian cancer (OC), with a focus on the methodological quality of these studies and the clinical utility of these proposed radiomics models.

**Methods:**

Original articles investigating radiomics in OC published in PubMed, Embase, Web of Science, and the Cochrane Library between January 1, 2002, and January 6, 2023, were extracted. The methodological quality was evaluated using the radiomics quality score (RQS) and Quality Assessment of Diagnostic Accuracy Studies 2 (QUADAS-2). Pairwise correlation analyses were performed to compare the methodological quality, baseline information, and performance metrics. Additional meta-analyses of studies exploring differential diagnoses and prognostic prediction in patients with OC were performed separately.

**Results:**

Fifty-seven studies encompassing 11,693 patients were included. The mean RQS was 30.7% (range − 4 to 22); less than 25% of studies had a high risk of bias and applicability concerns in each domain of QUADAS-2. A high RQS was significantly associated with a low QUADAS-2 risk and recent publication year. Significantly higher performance metrics were observed in studies examining differential diagnosis; 16 such studies as well as 13 exploring prognostic prediction were included in a separate meta-analysis, which revealed diagnostic odds ratios of 25.76 (95% confidence interval (CI) 13.50–49.13) and 12.55 (95% CI 8.38–18.77), respectively.

**Conclusion:**

Current evidence suggests that the methodological quality of OC-related radiomics studies is unsatisfactory. Radiomics analysis based on CT and MRI showed promising results in terms of differential diagnosis and prognostic prediction.

**Critical relevance statement:**

Radiomics analysis has potential clinical utility; however, shortcomings persist in existing studies in terms of reproducibility. We suggest that future radiomics studies should be more standardized to better bridge the gap between concepts and clinical applications.

**Graphical abstract:**

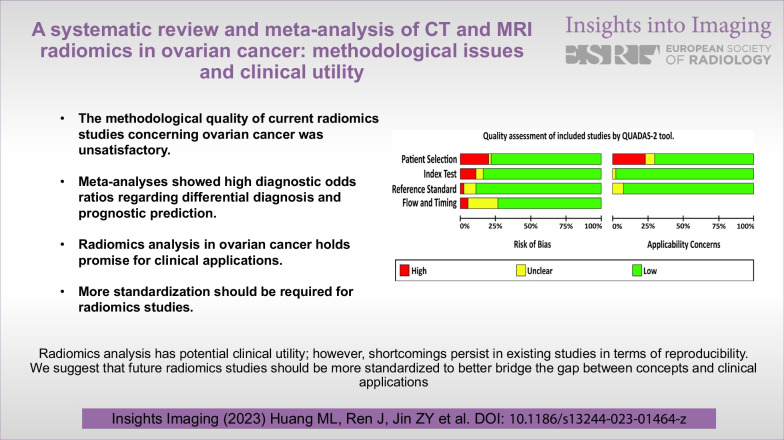

**Supplementary Information:**

The online version contains supplementary material available at 10.1186/s13244-023-01464-z.

## Key points

- The methodological quality of current radiomics studies concerning ovarian cancer was unsatisfactory.
- Meta-analyses showed high diagnostic odds ratios regarding differential diagnosis and prognostic prediction.
- Radiomics analysis in ovarian cancer holds promise for clinical applications.
- More standardization should be required for radiomics studies.

**Supplementary Information:**

The online version contains supplementary material available at 10.1186/s13244-023-01464-z.

## Introduction

Ovarian cancer (OC) is the most lethal gynecological cancer and the fifth-leading cause of cancer-related deaths among women; there were 313,959 newly diagnosed cases and 207,252 deaths worldwide in 2020 [1, 2]. The most recent cancer statistics report indicates that approximately 19,710 new cases of OC will be diagnosed in the USA in 2023, and 13,270 women will die from the disease [2]. The World Health Organization classification of tumors divides OC into dozens of pathological types [3]; furthermore, 70% of patients with OC are diagnosed at an advanced stage, leading to worse outcomes (their 5-year overall survival rates are 20–30% versus 80–95% for those diagnosed at early stages) [4–6]. Treatment involves surgery and chemotherapy and depends on the pathological type and International Federation of Gynaecology and Obstetrics stage [5, 7]. Notwithstanding advances in the diagnosis and treatment of OC, however, mortality rates have not changed appreciably in the last 30 years [2, 8–12]. This is partly due to the difficulty of early detection and the lack of effective therapeutic options for patients in advanced stages.

Computed tomography (CT) and magnetic resonance imaging (MRI) are essential for diagnosing and staging OC [5] and are invaluable for assessing chemotherapy response [13]. However, conventional imaging interpretations rely on the skills of radiologists, and variabilities among reports inevitably exist. Fortunately, medical imaging is advancing toward more standardized, specialized, and quantitative approaches, contributing to greater consistency and communication among radiologists. With this evolution, radiologists are shifting from conventional free-text reporting to structured reporting, enabling more accurate and efficient analysis of the extensive volumes of imaging data [14, 15]. Radiomics is a rapidly emerging field that quantitatively analyzes medical images utilizing artificial intelligence; based on high-throughput mining of quantitative image features, radiomics analysis generates unique markers that may be visually indiscernible yet can support clinical decision-making and increase diagnostic and prognostic accuracy [8, 16, 17].

In recent years, extensive studies using radiomics methods based on CT and MRI have linked quantitative image features to diagnosis, response evaluation, and prognostic prediction in patients with OC [8, 17, 18]. Nevertheless, it has remained difficult to assess the clinical value of radiomics in OC owing to the complexity of the methods and varying study designs. Therefore, we performed this study with two main aims: First, we evaluated the methodological quality of existing studies using the ‘quality of diagnostic accuracy studies-2’ (QUADAS-2) tool as well as the ‘radiomics quality score’ (RQS) [16, 19]. Second, we conducted a meta-analysis to determine the diagnostic performance of radiomics in patients with OC.

## Materials and methods

### Evidence acquisition protocol and registry

This systematic review was conducted according to the Preferred Reporting Items for Systematic Reviews and Meta-analysis (PRISMA) statement [20] (Additional file 1: Table S1). A review protocol is available through the International Prospective Register of Systematic Reviews (PROSPERO) (CRD 42022313519).

### Literature search and study selection

A structured search was performed independently by two reviewers (M.H. and J.R.) with 5 and 3 years of experience in gynecological imaging interpretation, respectively. Any disagreement was arbitrated by a third reviewer (Y.H.) with 12 years of experience, which was performed in 1.9% of the studies (8/418). The reviewers independently screened the titles, abstracts, and full texts of the extracted articles; uncertainties were discussed and resolved by consensus. Detailed search strategies and selection criteria are described in Additional file 1.

### Data extraction and methodological quality assessment

After selecting the relevant studies, the two reviewers developed a data extraction instrument (described in Additional file 1: Table S2). After independently reading the full text of each eligible article, they documented: (1) bibliographical information, (2) baseline study information (including study design, imaging technique parameters, and modeling information), and (3) model performance metrics. The extracted information was recorded using the Excel software (Microsoft Corp., Redmond, WA, USA). The methodological quality of the eligible articles was independently assessed by the two reviewers using the RQS (described in Additional file 1: Table S3) [16] and Quality Assessment of QUADAS-2 tools [19]. Each of the 16 key components identified by the RQS was rated, resulting in a total score ranging from − 8 to 36 points; these were converted to RQS percentages, with − 8 to 0 points defined as 0% and 36 points defined as 100% [16]. The QUADAS-2 tool comprises seven assessment items reflecting four domains: ‘patient selection’, ‘index test’, ‘reference standard’, and ‘flow and timing’. Each item was judged as “low,” “high,” or “unclear” based on responses to signaling questions on the risk of bias and applicability concerns [19]. A summed RQS rating calculated by averaging the scores of the two reviewers was determined for each study. For QUADAS-2 assessment, any disagreement was arbitrated with a third reviewer (H.Y.) to achieve a common appraisal for each item. To facilitate additional analysis, a final risk assessment was performed for each study as follows: studies in which all seven items were rated “low” were defined as “low risk”, studies in which at least one item was rated “high” were defined as “high risk”, and the remainder were defined as “unclear risk”.

### Data synthesis and analysis

Statistical analysis was conducted using the SPSS software version 25.0.0.0 (IBM Corp., Armonk, NY, USA), Review Manager (RevMan) version 5.3, and R (version 4.0.5; R Foundation for Statistical Computing) incorporating the ‘tidyverse’ packages. Categorical variables are presented as numbers and percentages, while continuous variables are presented as means and standard deviations or as medians and ranges. The inter-rater agreement for the RQS and QUADAS-2 was determined using Cohen’s kappa [21] and the ratio of agreements [21, 22]. An interclass correlation coefficient (ICC) of ≤ 0.4 was considered poor, 0.4–0.75 moderate, and > 0.75 good. Only training set performance metrics of the proposed radiomics models were recorded, even if validation sets existed, to maintain consistency among studies. Pairwise correlation analyses between methodological quality, baseline information, and performance metrics were conducted. Correlations between numeric variables were evaluated using linear regression analyses, while associations between numeric and categorical variables were assessed using unpaired Student’s *t*-tests or Mann–Whitney tests. The significance level *α* was 0.05, and statistical significance was set at a two-tailed *p*-value of < 0.05.

Meta-analyses were performed using the STATA software version 17.0 (StataCorp LP, College Station, TX, USA) with the ‘midas’ package when a sufficient number of studies attempted to address a similar question, and two-by-two tables could be extracted or reconstructed based on published data (the details are shown in Additional file 1). The sensitivity, specificity, positive likelihood ratio (PLR), negative likelihood ratio (NLR), and diagnostic odds ratio (DOR) with 95% confidence intervals (CIs) were calculated. Summary receiver operating characteristic (SROC) analysis was performed, and the areas under the curve (AUCs) were obtained to describe the diagnostic accuracy. If a particular study involved multiple models, only the radiomics model was selected. Heterogeneity was assessed using Cochrane’s *Q* test and Higgin’s inconsistency index (*I*^2^) test. Any heterogeneity was considered significant if the p-value on Cochran’s *Q* test was < 0.05, whereupon the random effects model was used. Higgins *I*^2^ values of < 25%, 25–50%, and > 50% were associated with low, moderate, and substantial heterogeneity, respectively. In cases of the latter, meta-regression was performed to explore the possible sources of heterogeneity. Deeks’ funnel plots were constructed to illustrate the risk of publication bias.

## Results

**Fig. 1:**
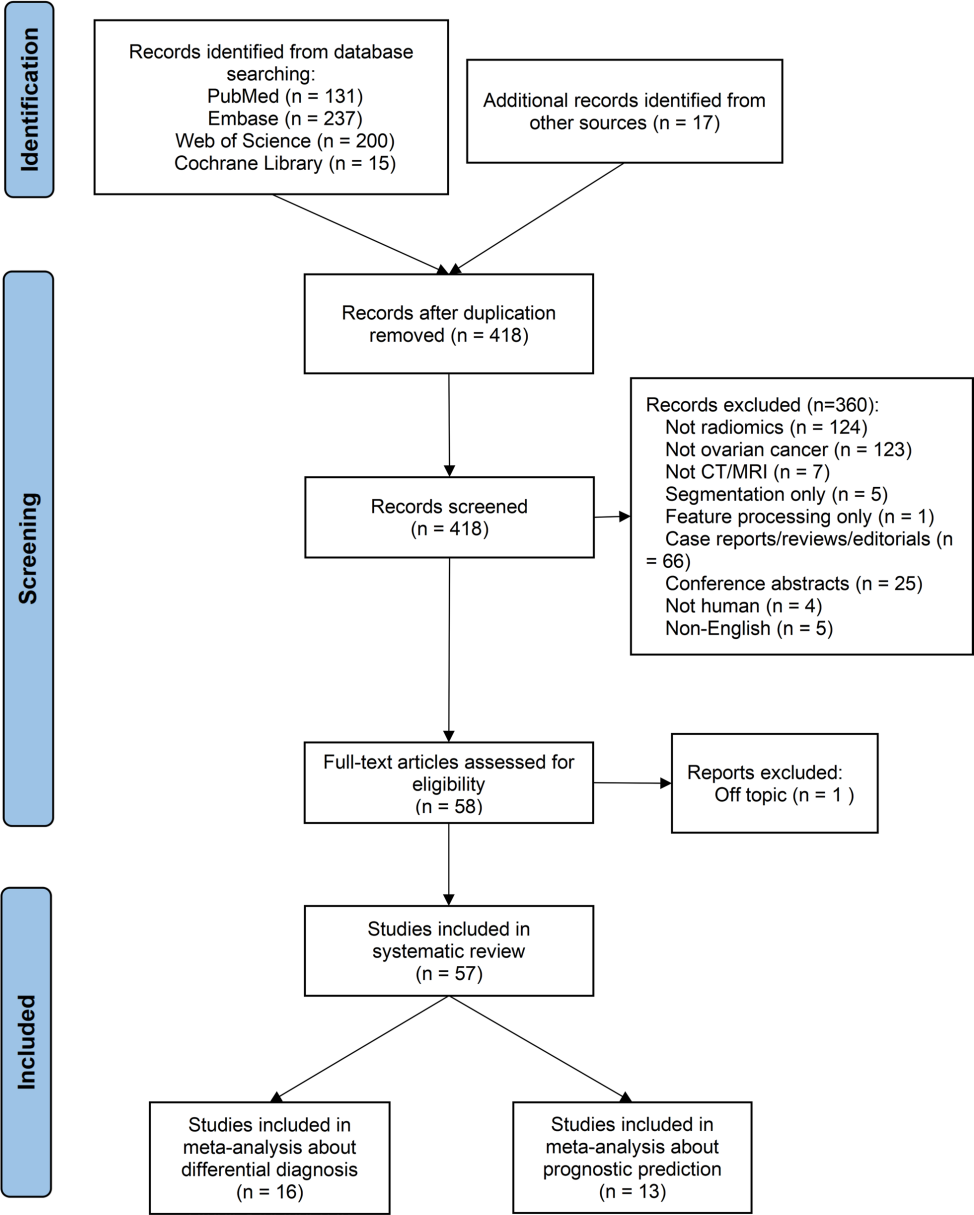
Flowchart of the study selection process for the systematic review and meta-analysis

The study selection process is illustrated in Fig. 1; 57 articles were ultimately included in the systematic review [23–79]. We divided these publications based on the studies’ main objectives into three categories: differential diagnosis (24/57, 42.1%), response evaluation (4/57, 7.0%), and prediction of prognosis (28/57, 49.1%). Additionally, one paper (1.8%) described both differential diagnosis and prognostic prediction. We found that assessments of differential diagnosis and prognostic prediction were both commonly performed; thus, 16 articles focusing on the differential diagnosis of OC [24–26, 28-32, 34, 35, 39, 40, 42, 44, 45, 47] and 13 that described studies on prognostic factors [52, 53, 55–57, 64, 66–68, 71–74] were subjected to separate meta-analyses.

### Study characteristics

There were between 28 and 1329 patients in each study; their median or mean ages ranged from 38.5 to 75 years. The studies’ baseline information and characteristics are shown in Tables 1 and 2, respectively. Nearly half of the studies were published in imaging journals (28/57, 49.1%); the first authors were mainly specialized in radiology (36/57, 63.2%) and most came from Asia (43/57, 75.4%). Thirty-seven studies used CT and 20 used MRI; most applied the manual segmentation method and three-dimensional analysis. According to the model classification method proposed by the TRIPOD statement [80], a plurality of the studies established developed models validated with random splitting of data (27/57, 47.4%), followed by developed models validated using exactly the same data (10/57, 17.5%) and separate data (9/57, 15.8%). Furthermore, using Zhong et al. method [22], 59.6% of the studies were classified as phase 0 owing to their lack of external validation. Two phase III studies were conducted without sufficient patients or lacked external validation; however, they retained their phase categories owing to their prospective designs. Detailed characteristics of each study’s population and proposed radiomics model are presented in Additional file 1: Tables S4 and S5.

**Table 1:** Baseline information of studies included in the systematic review

StudyID	Research question	#Pat	FIGO stages	Imaging modality	Study design	Outcome	Validation	Reference standard	RQS (%)
*Differential diagnosis*									
Zheng2022	Differentiating SBOTs and SMOTs	D = 125 IV = 31	NR	MRI (fs-T2WI, DWI)	R	SBOT or SMOT	Yes (internal)	HP	30.6
Zhang2022	Differentiating EOTs and MOTs	D = 187 IV = 99	NR	ceCT (VP)	R	EOT or MOT	Yes (internal)	HP	38.9
Xu2022	Differentiating: (1) BEOTs and EOCs; (2) type I and type II EOCs	D = 89 IV = 114 D = 48	30I/82II	MRI (DWI, ADC)	R	BEOTs or EOCs; (2) type I or type II EOCs	Yes (internal) No	HP	38.9
Wei2022	Differentiating benign and borderline EOTs	D = 309 IV = 78 EV = 30	NR	MRI (T2WI)	R	benign or borderline EOTs	Yes (internal and external)	HP	38.9
M.Wang2022	Differentiating HGSC and non-HGSC	D = 532 IV = 133	NR	ceCT	R	HGSC or non-HGSC	Yes (internal)	HP	34.8
Nagawa2022	Differentiating OTFGs and OGCTs	D = 53	17I/2II/2III	MRI (T2WI)	R	OTFG or OGCT	None	HP	13.9
LiuX2022	Differentiating BEOTs and MEOTs	D = 99 IV = 97	NR	MRI (T2, fs-T2)	R	BEOT or MEOT	Yes (internal)	HP	22.2
LiuP2022	Differentiating benign and malignant ovarian tumors	D = 96 IV = 39	NR	ceCT (AP)	R	Benign or malignant ovarian tumors	Yes (internal)	HP	29.2
LiS2022	Differentiating benign and malignant ovarian tumors	D = 99 IV = 41	NR	neCT	R	Benign or malignant ovarian tumors	Yes (internal)	HP	38.9
LiJ.1.2022	Differentiating benign and malignant ovarian tumors	D = 930 IV = 399	NR	ceCT (VP)	R	Benign or malignant ovarian tumors	Yes (internal)	HP	33.3
LiJ.2.2022	Differentiating type I and type II EOCs	D = 329 IV = 141	237I-II/233III-IV	ceCT (VP)	R	Type I or type II EOCs	Yes (internal)	HP	38.9
Zhu2021	Differentiating EOCs and NEOCs	D(IV) = 101	28I/14II/57III/2IV	neCT	R	EOC or NEOC	Yes (internal)	HP	38.9
YuXP2021	Differentiating SBOTs and SMOTs	D = 127 IV = 55	117I /65II	ceCT (AP, VP, EP)	R	SBOT or SMOT	Yes (internal)	HP	26.4
Ye2021	Differentiating BEOTs and FIGO stage I/II MEOTs	D = 62 IV = 26	I-II	MRI (T1, T2, DWI)	R	BEOT or FIGO stage I/II MEOT	Yes (internal)	HP	27.8
Song.1.2021	Differentiating benign, borderline, and malignant ovarian tumors	D + IV = 82	NR	MRI (DCE)	P	Benign, borderline, or malignant ovarian tumors	Yes (internal)	HP	55.6
Park2021	Differentiating benign and malignant ovarian tumors	D(IV) = 427	NR	ceCT	R	Benign or malignant ovarian tumors	Yes (internal)	Benign: (1) HP, (2) US or MRI, (3) stable after 2 years, or (4) resolved on subsequent imaging without treatment Malignant:HP	33.3
LiS2021	Differentiating benign and malignant ovarian tumors	D = 95 IV = 39 EV = 26	NR	CeCT (VP)	R	Benign or malignant ovarian tumors	Yes (internal and external)	HP	41.7
LiN2021	Differentiating OGCTs and OTCA–FTCA	D = 46	NR	MRI (T2)	R	OGCT or OTCA-FTCA	None	HP	11.1
Jian2021	Differentiating type I and type II EOCs	D = 144 IV = 75 EV = 75	NR	MRI (T1, T2, DWI, ADC)	R	Type I or type II EOC	Yes (internal and external)	HP	30.6
Hu2021	Differentiating POCs and SOCs	D = 76 IV = 34	NR	ceCT (neCT, AP)	R	POC or SOC	Yes (internal)	HP	30.6
An2021	Differentiating HGSC and non-HGSC	D = 163 IV = 42	44I/25II/106III/30IV	ceCT (VP)	R	HGSC or non-HGSC	Yes (internal)	HP	31.9
Qian2020	Differentiating type I and type II EOCs	D(IV) = 61	26I-II/35III-IV	MRI (fs-T2, DWI, DCE)	R	Type I or type II EOC	Yes (internal)	HP	44.4
Lupean2020	Differentiating benign and malignant ovarian cysts	D = 28	NR	MRI (T2)	R	Benign or malignant ovarian cysts	None	HP	5.6
Li2020	Differentiating BEOTs and MEOTs	D = 250 IV = 92 EV = 159	NR	MRI (T1, fs-T2, DWI, ADC)	R	BEOT or MEOT	Yes (internal and external)	HP	36.1
Zhang2019	Differentiating (1) benign and malignant ovarian tumors; (2) type I and type II EOC;	D = 195 IV = 85	I-IV	MRI (T1, T2, fs-T2, DWI)	R	(1) Benign or malignant ovarian tumors; (2) type I or type II EOC	Yes (internal)	HP, immunohistological staining	30.6
*Response evaluation*									
Rundo2022	Predicting response to neoadjuvant chemotherapy in HGSOC	D = 61 IV = 48	77IIIC/32IV	ceCT	R	Non-complete response (CRS1-2) or complete response (CRS3)	Yes (internal)	CRS	33.3
Zargari2018	Predicting tumor response to postsurgical chemotherapy in patients with advanced-stage ovarian cancer	D(IV) = 120	NR	ceCT (60 s)	R	Response to postsurgical chemotherapy	Yes (internal)	RECIST 1.1	30.6
Danala2017	Predicting tumor response to chemotherapy in ovarian cancer patients	D = 91	NR	ceCT (60 s)	R	Response to chemotherapy	None	RECIST 1.1	8.3
Qiu2016	Predicting early response of ovarian cancer patients to chemotherapy	D = 30	NR	ceCT (60 s, 5 min)	R	Early response to chemotherapy	None	RECIST 1.1	0.0
*Prognosis prediction*									
Wan2023	Predicting CCR5 expression level and survival	D = 57 IV = 89	NR	CT	R	CCR5 expression level, survival	Yes (internal)	CCR5 expression data from TCGA, OS	50.0
Wu2022	Predicting early recurrence in patients with HGSOC	D = 74 IV = 36	33I-II/77III-IV	ceCT (AP, VP)	R	Cancer recurrence	Yes (internal)	PFS	33.3
WangT2022	Predicting EOC prognosis	D = 130 IV = 56	85I/16II/76III/9IV	MRI (T1, T2, DWI, CE-T1)	R	Survival	Yes (internal)	DFS	33.3
Lu2022	Predicting residual tumor in patients with HGSOC	D = 106 IV = 22	5IIIA/5IIIB/82IIIC/36IV	MRI (T2WI, DWI, ADC)	R	RT status	Yes (internal)	RT status from operative reports	38.9
LiC2022	Predicting recurrence in patients with HGSOC	D = 98 IV = 43	19I-II/122III-IV	MRI (fs-T2WI, DWI, T1WI + C)	R	Postoperative recurrence	Yes (internal)	DFS	33.3
Hu2022	Predicting OS and DFS in patients with HGSOC	D = 95 IV = 90 EV = 32	32I/43II/121III/8IV	ceCT (VP)	R	Survival	Yes (internal and external)	OS, DFS	30.6
Hong2022	Predicting survival in patients with serous ovarian cancer	D = 80 EV = 39	13I/11II/66III/29IV	ceCT (VP)	R	Survival	Yes (external)	OS	27.8
Gao2022	Predicting the expression of PD-1 and OS in OC patients	PDCD1: D + IV = 57 Survival: IV = 89	PDCD1: NR Survival: 47II-III/42IV-unknown	CT	R	PD-1 expression, survival	Yes (internal)	PD-1 expression status from TCGA-OV, OS	38.9
Fotopoulou2022	Validating the prognostic value of RPV in patients with HGSOC	V = 547	15I/30II/2671III/227IV/8unknown	ceCT (VP)	R	Survival, operability	Yes (external)	PFS, OS, macroscopic tumor clearance	36.1
Feng2022	Predicting hypoxia pattern in patient prognostication	D = 40 IV = 19	NR	ceCT (AP)	R	Hypoxia pattern	Yes (internal)	nine-gene panel	27.8
Boehm2022	Predicting risk stratification of HGSOC	D = 298 IV = 40	NR	ceCT (VP)	R	Survival	Yes (internal)	OS, PFS	34.7
Avesani2022	Predicting BRCA mutation and PFS in patients with HGSOC	D = 152 EV = 66	5I/17II/1471III/45IV/4unknown	ceCT (VP)	R	BRCA mutation, Survival	Yes (external)	BRCA mutation status, PFS	36.1
YuXY2021	Predicting peritoneal carcinomatosis in EOC patients before surgery	D = 86	NR	MRI (fs-T2, DWI, DCE)	R	Peritoneal carcinomatosis	None	HP	19.4
Yi2021	Predicting platinum resistance for OC treatment	D = 71 IV = 31	8II/80III/14IV	ceCT (neCT, VP)	R	Platinum resistance	Yes (internal)	6-month PFS	36.1
Song.2.2021	Predicting peritoneal metastasis in ovarian cancer	D = 54 IV = 35	NR	MRI (T2, fs-T2, DWI)	P	Peritoneal metastasis	Yes (internal)	HP	61.1
Liu2021	Predicting BRCA gene mutation status in patients with EOC	D = 106	NR	ceCT (AP, VP, DP)	R	BRCA mutation	Yes (internal)	NGS genetic testing	36.1
LiM2021	Predicting BRCA gene mutation status in patients with advanced EOC	95 (D:IV = 7:3)	32III/63IV	ceCT (AP, VP, DP)	R	BRCA mutation	Yes (internal)	NGS genetic testing	36.1
LiH.1.2021	Predicting RFS in patients with advanced HGSOC	D(IV) = 117	III-IV	MRI (CE-T1, T2)	R	Survival	Yes (internal)	RFS	27.8
LiH.2.2021	Predicting residual disease in patients with advanced HGSOC	D = 160 IV = 57	III-IV	MRI (CE-T1, T2)	R	Residual disease	Yes (internal)	R0 resection	23.6
Chen.1.2021	Predicting early recurrence in patients with HGSOC	D = 179 IV = 77	55 I-II/201 III-IV	ceCT (VP)	R	Early recurrence	Yes (internal)	PFS	33.3
Chen.2.2021	Predicting preoperative LN metastasis in patients with HGSOC	D = 179 IV = 77	NR	ceCT (VP)	R	Preoperative LN metastasis	Yes (internal)	HP	41.7
Ai2021	Predicting metastatic status of OC patients	D = 70 IV = 31	29I/14II/57III/1IV	neCT	R	Preoperative metastasis	Yes (internal)	HP	22.2
Veeraraghavan2020	Predicting PFS and platinum resistance in patients with HGSOC	D(IV) = 40 EV = 35	58III/17IV	ceCT	R	Survival, platinum resistance	Yes (internal and external)	PFS	36.1
Wei2019	Predicting risk for postoperative advanced HGSOC recurrence	D = 50 IV = 50 EV = 42	112III/30IV	ceCT (neCT, AP, VP)	R	Postoperative recurrence	Yes (internal and external)	PFS	38.9
Meier2019	Predicting survival and BRCA mutation status in patients with HGSOC	D = 88	NR	ceCT (VP)	R	Survival, BRCA mutation	None	OS, PFS, BRCA mutation status	0.0
Lu2019	Predicting prognostic- and molecular-phenotypes of EOC	D = 136 IV = 77 EV = 70	53I-II/223III-IV/18 unknown	ceCT	R	Survival, molecular-phenotypes	Yes (internal and external)	PFS, OS	40.3
Zhang2019*	Predicting survival among EOC patients	D = 195 IV = 85	I-IV	MRI (T1, T2, fs-T2, DWI)	R	Survival	Yes (internal)	DFS	30.6
Rizzo2018	Predicting residual tumor at surgery and the risk of PD12 in OC patients	D = 101	11II/66III/24IV	ceCT (VP)	R	Residual tumor, risk of PD12	None	RT, PD12	8.3
Vargas2017	Predicting outcomes in patients with HGSOC	D = 38	23III/15IV	ceCT (70 s)	R	Surgical resection, survival	None	Surgical resection status, molecular analysis, OS	12.5

One study discussed two topics and was described twice, which was marked with “*”

*BEOT* borderline epithelial ovarian tumor, *MEOT* malignant epithelial ovarian tumor, *EOC* epithelial ovarian cancer, *NEOC* non-epithelial ovarian cancer, *SBOT* serous borderline ovarian tumor, *SMOT* serous malignant ovarian tumor, *MOT* metastatic ovarian tumor, *EOT* epithelial ovarian tumor, *OTFG* ovarian granulosa cell tumor, *OGCT* ovarian granulosa cell tumor, *OTCA–FTCA* thecoma–fibrothecoma, *POC* primary ovarian cancer, *SOC* secondary ovarian cancer, *HGSC* high-grade serous carcinoma, *HGSOC* high-grade serous ovarian carcinoma, *OC* ovarian cancer, *RPV* Radiomic Prognostic Vector, *D* development, *IV* internal validation, *EV* external validation, *V* validation, *AP* arterial phase, *VP* venous phase, *EP* equilibrium phase, *DP* delay phase, *R* retrospective, *P* prospective, *CRS* chemotherapy response score, *LN* lymph node, *PD12* disease progression within 12 months, *HP* histopathology, *DFS* disease-free survival, *PFS* progression-free survival, *RFS* recurrence-free survival, *OS* overall survival, *RT* residual tumor, *NRnot* reported, *NA* not applicable

**Table 2:** Characteristics of included studies

Characteristics	No. of studies (%)
*Journal speciality*	
Imaging	28/57 (49.1%)
Clinical	20/57 (35.1%)
Generalist	7/57 (12.3%)
Medical physics	2/57 (3.5%)
Specialty of the 1st author*	
Radiologist	36/57 (63.2%)
Radiotherapist	1/57 (1.8%)
Clinician	11/57 (19.3%)
Physicist, Computer scientist	9/57 (15.8%)
*Geographical origin of the study*	
Asia	43/57 (75.4%)
Europe	6/57 (10.5%)
North America	8/57 (14.0%)
*Imaging modality*	
CT	37/57 (64.9%)
MRI	20/57 (35.10%)
Study objectives**	
Differential diagnosis	25/57 (43.9%)
Response evaluation	4/57 (7.0%)
Prognosis prediction	29/57 (50.9%)
*Segmentation method*	
Manual	53/57 (93.0%)
Semi-automatic	4/57 (7.0%)
*Publication of negative results*	
No	55/57 (96.5%)
Yes	2/57 (3.5%)
*Type of ROI*	
2D	11/57 (19.3%)
3D	43/57 (75.4%)
2D&3D	3/57 (5.3%)
*Model type*	
Type 1a: developed model validated with exactly the same data	10/57 (17.5%)
Type 1b: developed model validated with resampling data	6/57 (10.5%)
Type 2a: developed model validated with randomly splitting data	27/57 (47.4%)
Type 2b: developed model validated with non-randomly splitting data	4/57 (7.0%)
Type 3: developed model validated with separate data	9/57 (15.8%)
Type 4: validation only	1/57 (1.8%)
Phase classification***	
Discovery science: experimental	10/57 (17.5%)
Phase 0: < 100 patients; retrospective; internal validation Or > 100 patients; retrospective; internal validation	34/57 (59.6%)
Phase I: < 100 patients; retrospective; external validation	2/57 (3.5%)
Phase II: > 100 patients; retrospective; external validation	9/57 (15.8%)
Phase III: > 100 patients; prospective; external validation	2/57 (3.5%)
Phase IV: real world	0/57

*Thirteen studies claimed joint first author, the author whose name was written first was assessed

**One study discussed two topics

***Two studies were classified as phase III due to prospective design, although they were conducted with less than 100 patients and without external validation

### Methodological quality assessment

The 57 studies had a mean RQS of 12 (interquartile range 10–14), with RQS values ranging from − 4 to 22. The mean percentage RQS was 30.7%, with a maximum of 61.1%. The average ICC for the RQS was 0.80 (95% confidence interval (CI) 0.69–0.91), i.e., a ‘good’ rating. The average rating and inter-rater agreement per RQS component are shown in Table 3. In most studies, the lack of reproducibility and repeatability analysis of imaging features (e.g., phantom study and imaging at multiple time points), insufficient cost-effectiveness analyses, and inadequate access to the data led to a low RQS. Additionally, biological correlation and cutoff analyses were seldom performed in the aftermath, and the rated validation score (2 [2]) was suboptimal. Discrimination statistics to assess model performance were available for all studies; 21 of them also reported calibration statistics. Fifty-five studies (96.5%) were retrospective analyses, while only two (3.5%) were based on prospectively acquired data. The RQS assessments by each reviewer (M.H. and J.R.) are shown in Additional file 1: Table S6.

**Table 3:** Average rating and inter-rater agreement per component of RQS

No.	RQS item	Range	Average	Median (range)	Inter-rater agreement	
					ICC	95%CI
1	Image protocol quality	0–2	0.89	1 (0–2)	0.90	0.76–1.00
2	Multiple segmentations	0–1	0.63	1 (0–1)	1.00	1.00–1.00
3	Phantom study on all scanners	0–1	0.05	0 (0–1)	1.00	1.00–1.00
4	Imaging at multiple time points	0–1	0	0 (0–0)	1.00	1.00–1.00
5	Feature reduction or adjustment for multiple testing	− 3 to 3	2.79	3 (− 3 to 3)	1.00	1.00–1.00
6	Multivariable analysis with non-radiomics features	0–1	0.72	1 (0–1)	0.91	0.80–1.00
7	Detect and discuss biological correlate	0–1	0.23	0 (0–1)	0.90	0.77–1.00
8	Cutoff analyses	0–1	0.13	0 (0–1)	0.77	0.52–1.00
9	Discrimination statistics	0–2	1.42	0 (0.5–2)	0.72	0.54–0.90
10	Calibration statistics	0–2	0.42	0 (0–2)	1.00	1.00–1.00
11	Prospective study registered in a trial database	0–7	0.25	0 (0–7)	1.00	1.00–1.00
12	Validation	− 5 to 5	1.39	2 (− 5 to 5)	0.94	0.85–1.00
13	Comparison to 'gold standard'	0–2	1.26	2 (0–2)	0.93	0.82–1.00
14	Potential clinical utility	0–2	0.70	0 (0–2)	1.00	1.00–1.00
15	Cost-effectiveness analysis	0–1	0	0 (0–0)	1.00	1.00–1.00
16	Open science and data	0–4	0.15	0 (0 ≥ 2)	0.92	0.78–1.00
	Total points: − 8 to 0 = 0%, 36 = 100%	0–100%	11.04 = 30.7%	12 (− 4 to 22)	0.80	0.69–0.91

*CI* confidence interval, *ICC* interclass correlation coefficient, *RQS* Radiomics Quality Score

**Fig. 2:**
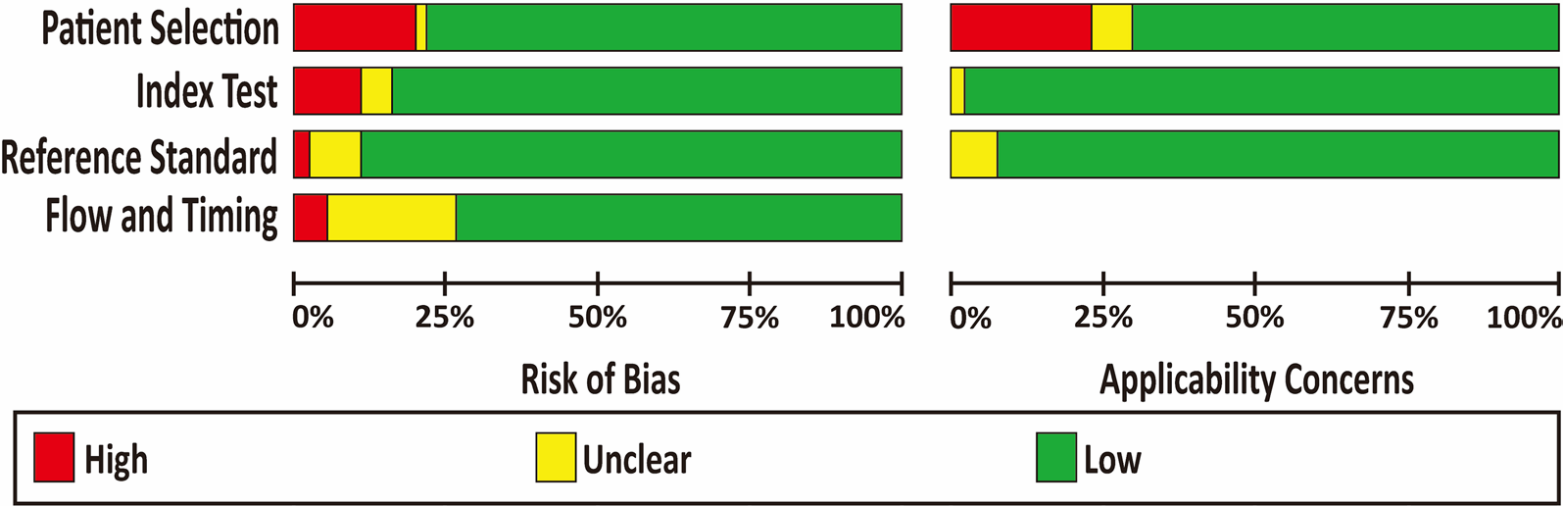
Quality assessment of included studies by QUADAS-2 tool. The authors’ judgments for each domain of each included study were reviewed. The proportion of included studies that indicated low, unclear, high risk, and applicability concerns were shown in green, yellow and gray, respectively

The summarized and individual QUADAS-2 results are presented in Fig. 2 and Additional file 1: Table S7. A high risk of publication bias (19.3%) and applicability concerns (22.8%) were observed in terms of patient selection, mainly because of inappropriate exclusion. Twelve studies (21.1%) were regarded as having an unclear risk of bias in flow and timing, as they did not provide sufficient information regarding the interval between index tests and reference standards. Complete agreement between the two reviewers concerning the seven QUADAS-2 items ranged from 80.7 to 96.5%.

### Pairwise correlation analysis of methodological quality, baseline information, and performance metrics

**Fig. 3:**
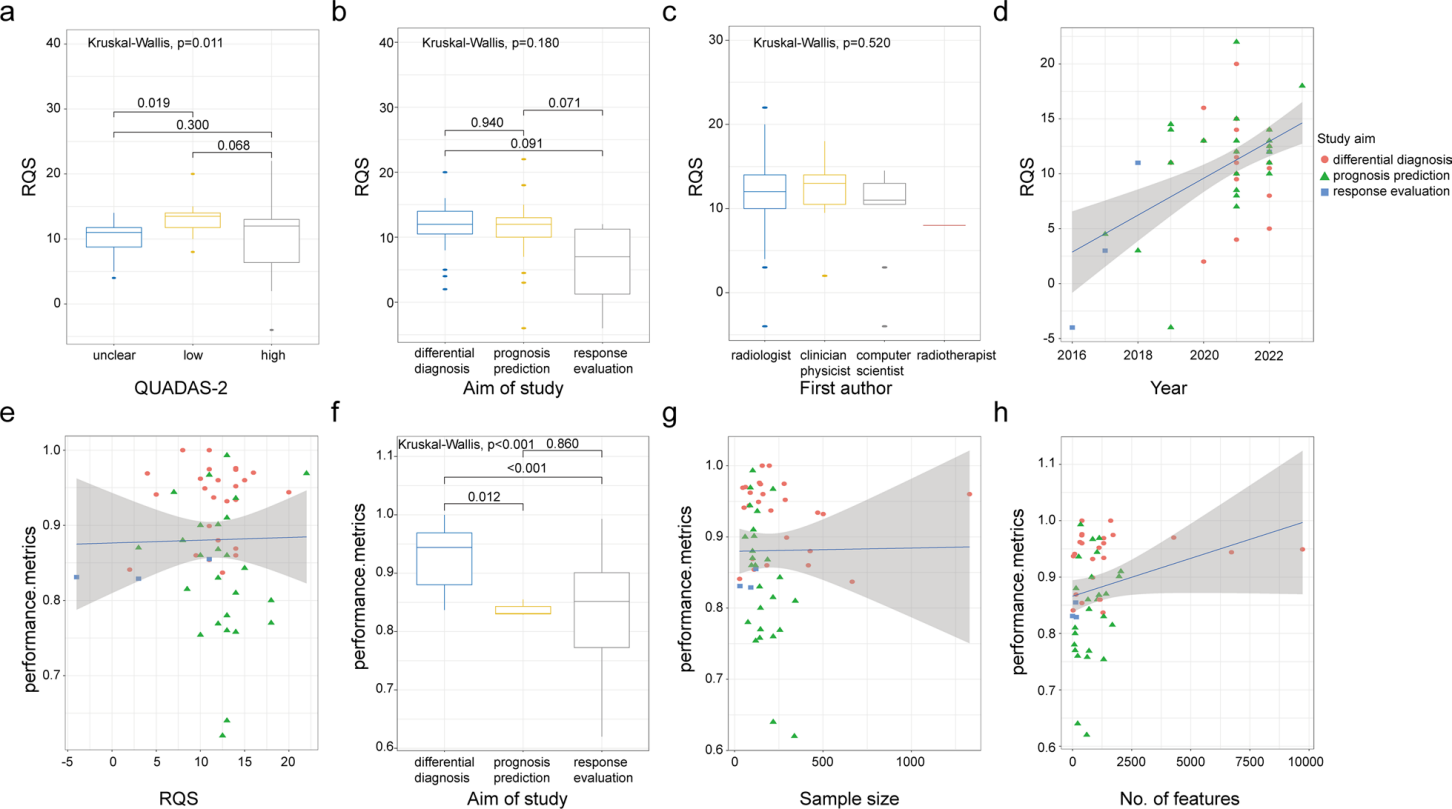
Correlations between radiomics quality score (RQS), performance metrics and baseline information. **a**–**d** Correlation between RQS and QUADAS-2, study aim, first author, and publication year. The vertical dashed line corresponds to the year of publication of the RQS. **e**–**h** Correlation between performance metrics and RQS, study aim, number of patients, and number of features. Each point corresponds to a study. The regression line and its 95% confidence interval are shown in gray with adjusted *R*^2^ and *p*-value

The pairwise correlation analysis results are presented in Fig. 3. One article that discussed both differential diagnosis and prognostic prediction was analyzed twice as two separate studies. The RQS values were significantly different between studies with different QUADAS-2 risk assessments (*p* = 0.011). Importantly, studies deemed low risk had a higher mean RQS than those with high or unclear risk; the difference between low-risk and unclear-risk studies was significant (*p* = 0.002). The study aim (*p* = 0.180) and specialty of the first author (*p* = 0.520) did not influence the RQS rating. Studies published more recently received significantly higher scores (adjusted *R*^2^ = 0.264, *p* < 0.001).

The performance metrics were represented by the AUC in 49 studies (86.0%) and by the c-index in three (5.3%); they were missing in the remaining five (8.8%). Two of the studies explored two radiomics models with different aims; hence, their performance metrics were documented separately. The best radiomics model used in each study had an AUC or c-index value between 0.620 and 1.000. Moreover, the performance of the radiomics model was closely related to the study aim (*p* < 0.001); performance was significantly better in studies exploring differential diagnosis than in those evaluating response (*p* = 0.012) or predicting prognosis (*p* < 0.001). No significant correlation was found between performance metrics and RQS (adjusted *R*^2^ = − 0.019, *p* = 0.892), sample size (adjusted *R*^2^ = − 0.019, *p* = 0.938), or number of features extracted (adjusted *R*^2^ = 0.046, *p* = 0.068).

### Meta-analysis

**Fig. 4:**
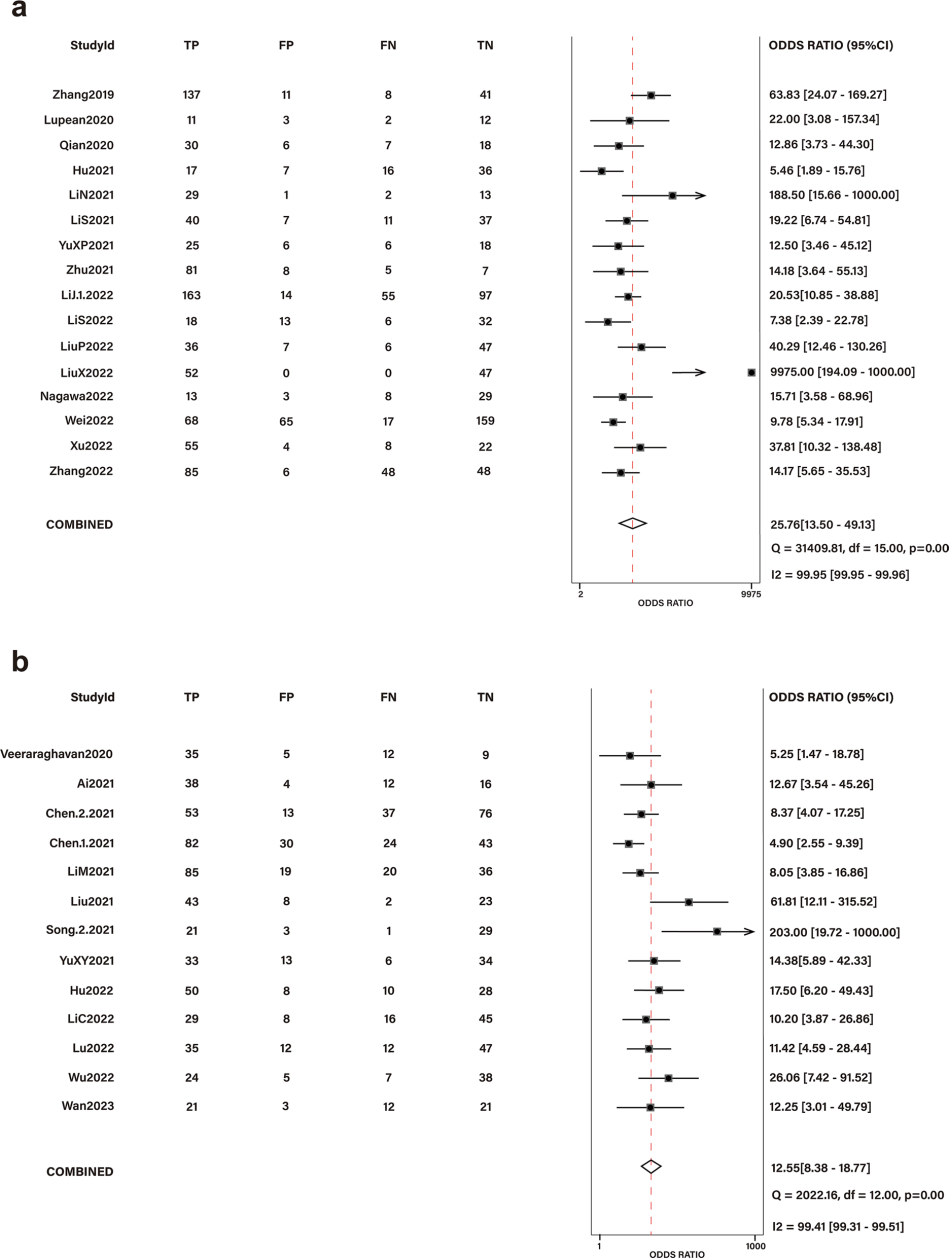
Forrest plot of the effect size calculated as diagnostic odds ratio for studies discussing the diagnostic accuracy of radiomics models in the **a** differential diagnosis; **b** prognosis prediction of ovarian tumors. The numbers are pooled estimates with 95% CIs in parentheses; horizontal lines indicate 95% CIs. TP, FP, FN, and TN were defined according to the original articles' descriptions

Sixteen studies that focused on differential diagnosis and 13 that investigated prognostic prediction were subjected to a meta-analysis. For studies of differential diagnosis, the pooled diagnostic odds ratio (DOR) was 25.76 (95% CI 13.50–49.13) (Fig. 4a); the pooled sensitivity, specificity, positive likelihood ratio (PLR), and negative likelihood ratio (NLR) were 0.84 (95% CI 0.76–0.89), 0.83 (95% CI 0.77–0.88), 5.00 (95% CI 3.58–6.97), and 0.19 (95% CI 0.13–0.30), respectively (Additional file 1: Figs. S1a and Fig. S2a). The SROC curve suggested good diagnostic performance with an AUC of 0.90 (95% CI 0.87–0.92) (Additional file 1: Fig. S3a). For studies analyzing prognostic prediction, the pooled DOR, sensitivity, specificity, PLR, and NLR were 12.55 (95% CI 8.38–18.77), 0.78 (95% CI 0.71–0.83), 0.78 (95% CI 0.72–0.82), 3.59 (95% CI 2.80–4.59), and 0.29 (95% CI 0.22–0.37), respectively (Fig. 4b, Additional file 1: Figs. S1b, and S2b). The AUC calculated from the SROC curve was 0.85 (95% CI 0.81–0.88), indicating good performance of the prognostic prediction models (Additional file 1: Fig. S3b). Per Deeks’ funnel plots, the likelihood of publication bias was low for differential diagnosis studies (*p* = 0.760) but high for prognostic prediction studies (*p* = 0.040) (Additional file 1: Fig. S4).

Cochrane’s *Q* test (*p* < 0.001 and *p* < 0.001) and Higgin’s *I*^2^ test (*I*^2^ = 94% and *I*^2^ = 91%) indicated high heterogeneity; therefore, a meta-regression analysis was conducted to identify its source (Table 4 and Additional file 1: Fig. S5). The number of patients was significantly associated with heterogeneity in specificity (*p* < 0.001) for differential diagnosis studies and contributed to heterogeneity in sensitivity (*p* < 0.001), specificity (*p* < 0.001), and the AUC (*p* = 0.04) in prognostic prediction studies. Additionally, the imaging method (CT vs. MRI) influenced heterogeneity in both sensitivity (*p* < 0.001 for differential diagnosis and *p* < 0.01 for prognostic prediction studies) and specificity (*p* < 0.01 for both study types). The heterogeneity of sensitivity and specificity in the differential diagnosis subgroup was associated with the type of region of interest (*p* = 0.04 for sensitivity and *p* < 0.01 for specificity). In the prognostic prediction subgroup, however, the region of interest type influenced AUC (*p* = 0.03), sensitivity (*p* < 0.001), and specificity (*p* < 0.01). Meanwhile, the type of features only contributed to heterogeneity in specificity (*p* < 0.01 for both differential diagnosis and prognostic prediction).

**Table 4:** The results of meta-regression analysis of studies about differential diagnosis and prognosis prediction of OC

Covariates	Subgroup	No. of studies	Sensitivity [95%CI]	*P*_SEN_	Specificity [95%CI]	*P*_*S*PE_	*P*
*Differential diagnosis*							
Number of patients	< 100	11	0.83 [0.75–0.92]	0.15	0.86 [0.80–0.91]	< 0.001	0.42
	≥ 100	5	0.84 [0.74–0.95]		0.78 [0.69–0.88]		
Imaging modality	MRI	8	0.89 [0.82–0.95]	< 0.001	0.85 [0.78–0.93]	< 0.01	0.17
	CT	8	0.78 [0.68–0.88]		0.81 [0.73–0.89]		
Type of ROI	2D	7	0.85 [0.76–0.95]	0.04	0.84 [0.75–0.92]	< 0.01	0.92
	3D	9	0.83 [0.74–0.92]		0.83 [0.76–0.90]		
Type of features	Low-order	12	0.83 [0.75–0.91]	0.26	0.84 [0.78–0.90]	< 0.01	0.77
	High-order	4	0.86 [0.75–0.97]		0.81 [0.71–0.91]		
*Prognosis prediction*							
Number of patients	< 100	9	0.79 [0.73–0.85]	< 0.001	0.82 [0.76–0.87]	< 0.001	0.04
	≥ 100	4	0.73 [0.64–0.82]		0.73 [0.64–0.82]		
Imaging modality	MRI	5	0.79 [0.71–0.88]	< 0.01	0.79 [0.70–0.87]	< 0.01	0.85
	CT	8	0.76 [0.69–0.84]		0.78 [0.71–0.85]		
Type of ROI	2D	4	0.84 [0.77–0.92]	< 0.001	0.82 [0.74–0.91]	< 0.01	0.03
	3D	9	0.74 [0.68–0.80]		0.76 [0.69–0.83]		
Type of features	Low-order	6	0.74 [0.65–0.82]	0.09	0.80 [0.70–0.85]	< 0.01	0.46
	High-order	7	0.81 [0.74–0.88]		0.77 [0.70–0.85]		

## Discussion

Our systematic review found that the methodological quality of CT and MRI radiomics for patients with OC was relatively low, while our meta-analysis revealed that radiomics has promising potential in discriminating between OC subtypes and predicting patient prognosis.

Several narrative reviews described the prospects of applying radiomics in OC [8, 17, 81, 82]. Rizzo et al.’s systematic review [83] included six studies that lacked RQS ratings and meta-analyses, whereas that by Ponsiglione et al. [18] used the RQS to evaluate the methodological quality of studies involving CT-, MRI-, ultrasonography-, or positron emission tomography-based radiomics in ovarian disorders published as of November 2021. The field of radiomics is in a period of rapid growth; 42.1% of the studies we analyzed were published in 2022 or 2023. Aside from the RQS, QUADAS-2 was applied to assess potential publication bias and applicability concerns. While previously published reviews usually describe only qualitative analyses, we performed quantitative evaluation of the effect of radiomics models by conducting a meta-analysis; our mean RQS (30.7%) was acceptable when compared to those published previously (5.6–36.1%) [18, 22, 84-87]. However, the overall scientific quality was still unsatisfactory, given that the assessment values were considerably below 100%. The main reasons for the low RQS ratings, as in previous analyses [22, 86, 88], included low levels of feature robustness, insufficient model assessment, and lack of concern for clinical applications.

The primary challenge for feature robustness was the high variability in radiomics features; few of the publications we analyzed described phantom studies [74, 77, 78], multiple time-point imaging, or automatic segmentation [49–51]. Regarding model assessment, radiomics studies usually included discrimination statistics; however, cutoff analyses and calibration statistics were often neglected, thus complicating the risk evaluation of overly optimistic results and accuracy of model predictions. Classification indexes including sensitivity and specificity, which are critical for quantitative analysis, were lacking in some studies. Our results suggest the need for performing additional technical validation before considering radiomics models for clinical applications.

Although most studies involved internal validation (which is indispensable for the clinical translation and broad application of radiomics models), the absence of external validation at several centers undermined the credibility and generalizability of the models. Very few prospective studies [37, 66], which are considered to have a high level of evidence, were performed. Furthermore, analysis of cost-effectiveness was absent from all our included studies, and the lack of decision curve analysis in approximately two-thirds of them also obscured the applicability of the models to clinical settings. As such, technical and clinical validations remain critical unmet requirements for incorporating radiomics analysis into clinical applications.

The importance of open science is emphasized in the RQS ratings to ensure the transparency and reproducibility of research findings, but such access was lacking in our studies. As such, we suggest that researchers should at least include the computed numerical values of any investigated features in their publications.

In terms of correlations between the RQS and original variables, there was a significant relationship between RQS and publication year. More modern studies presumably have a larger sample size, collect data from multiple centers, use a prospective design, and/or apply more strict inclusion and exclusion criteria, thereby contributing to a higher RQS. Additionally, a higher RQS was significantly correlated with low-level risk according to QUADAS-2, which may support using the former to improve research quality. We also found that studies exploring differential diagnosis had significantly higher performance metrics, whereas studies of response evaluation or prognostic prediction may be more affected by non-relevant factors, such as lifestyle and other medications, given that they have longer follow-up periods. Contrary to our expectation, we did not find a significant relationship between performance metrics and the number of features extracted, which could be explained by the heterogeneity in feature selection and modeling.

The results of our meta-analysis were promising; the pooled AUCs reached 0.90 for differential diagnosis and 0.85 for prognostic prediction, indicating a relatively high accuracy in categorizing different pathological subtypes and predicting the prognoses of patients with OC. According to Cochrane’s Q and Higgin’s I^2^ tests, studies included in the meta-analysis had high levels of heterogeneity, which a meta-regression analysis attributed to the influence of patient number, imaging modality, region of interest, and feature type.

Our study had some limitations. First, most included articles did not report the numbers of true/false positives or true/false negatives, and our calculation of these data from the available information might have introduced some errors. Second, studies predicting chemotherapy response were not included in the meta-analysis owing to insufficient data. Third, studies involved in the meta-analysis showed high heterogeneity; although we found significant correlations between such heterogeneity and certain factors, the latter may not have sufficiently explained the former. Fourth, the prognostic prediction studies included in our meta-analysis had a significant risk of publication bias, likely because we only included English-language articles and also because half of the studies comprised small sample sizes; reluctance to publish negative data may also have been a factor.

In conclusion, radiomics analysis showed promise in terms of overcoming some current obstacles in determining differential diagnosis, chemotherapy response, and prognosis in patients with OC. Pairwise correlation analysis revealed a significant relationship between RQS and QUADAS-2 result or publication year, as well as between performance metrics and study aims. Additionally, our meta-analysis demonstrated the suitability of radiomics analysis for discriminating between various subtypes of OC and identifying prognostic factors through quantitative analysis. Given the generally low RQS ratings of all the included studies, the methodological quality of radiomics studies involving OC is lower than desirable; hence, more high-level evidence is required to develop effective radiomics models.

## Supplementary Information

**Additional file 1**. Electronic Supplementary Materials.

## Data Availability

All data generated or analyzed during this study are included in this published article [and its Additional files].

## Abbreviations

AUC: Area under the curve
CI: Confidence interval
DOR: Diagnostic odds ratio
ICC: Interclass correlation coefficient
NLR: Negative likelihood ratio
OC: Ovarian cancer
PLR: Positive likelihood ratio
PRISMA: Preferred Reporting Items for Systematic Reviews and Meta-analysis
PROSPERO: International Prospective Register of Systematic Reviews
QUADAS-2: Quality Assessment of Diagnostic Accuracy Studies 2
RQS: Radiomics quality score
SROC: Summary receiver operating characteristic
