# Supplementary material for: A systematic review and meta-analysis of CT and MRI radiomics in ovarian cancer: methodological issues and clinical utility
Source: Insights Imaging. 2023 Jul 3;14:117. doi: 10.1186/s13244-023-01464-z (PMC10317928; doi:10.1186/s13244-023-01464-z)
Supplement: Supplementary file 1 — Additional file 1. Electronic Supplementary Materials. [file 13244_2023_1464_MOESM1_ESM.pdf]

A systematic review and meta-analysis of CT and MRI radiomics in ovarian cancer: methodological issues and clinical utility

ELECTRONIC SUPPLEMENTARY MATERIAL

List of Supplementary Materials

Supplementary study search strategy and selection criteria

Supplementary study bibliographical information and model performance metrics

Supplementary Table S1. PRISMA diagnostic accuracy checklists

Supplementary Table S2. Data collection instrument definitions

Supplementary Table S3 RQS checklist

Supplementary Table S4. Characteristics of study population in each study

Supplementary Table S5. Detailed radiomics models characteristics in each study

Supplementary Table S6. Individual RQS ratings (MH / JR) and average rating per item

Supplementary Table S7. QUADAS-2 assessment for each study

Supplementary Table S8 The results of meta-regression analysis of studies about differential diagnosis and prognosis prediction of OC.

Supplementary Figure S1. Forrest plot of pooled sensitivity and specificity of radiomics models in A) differentiation diagnosis, B) prognosis prediction.

Supplementary Figure S2. Forrest plot of pooled positive likelihood ratio and negative likelihood ratio of radiomics models.

Supplementary Figure S3. HSROC curve of the performance for radiomics models.

Supplementary Figure S4. Deeks funnel plot of studies included in the meta-analysis.

Supplementary Figure S5. Univariable meta-regression analysis based on patient, imaging modality, type of ROI and type of features.

Supplementary study search strategy and selection criteria

1. Study search strategy

1.1 PubMed search strategy

Available via https://pubmed.ncbi.nlm.nih.gov

Preliminary search date: 05 Jan 2023

Articles retrieved:131

Formal search date: 06 Jan 2023

Articles retrieved: 131

(textural OR radiomics OR radiomic OR texture OR machine learning) AND (ovary OR ovarian OR Ovaries) AND (computed tomography OR CT OR magnetic resonance OR MRI OR MR)

Filters: from 2002 - 2023

1.2 Embase search strategy

Available via www.embase.com

Preliminary search date: 05 Jan 2023

Articles retrieved:237

Formal search date: 06 Jan 2023

Articles retrieved: 237

#1 textural OR radiomics OR radiomic OR texture OR (machine AND learning)

#2 'ovary'/exp OR ovary OR ovarian OR 'ovaries'/exp OR ovaries

#3 ct OR (computed AND tomography) OR (magnetic AND resonance) OR mri OR mr

#4 #1 AND #2 AND #3

#5 #4 AND (2004:py OR 2006:py OR 2007:py OR 2008:py OR 2009:py OR 2010:py OR 2011:py OR 2012:py OR 2013:py OR 2014:py OR 2015:py OR 2016:py OR 2017:py OR 2018:py OR 2019:py OR 2020:py OR 2021:py OR 2022:py OR 2023:py)

(textural OR radiomics OR radiomic OR texture OR OR (machine AND learning)) AND ('ovary'/exp OR ovary OR ovarian OR 'ovaries'/exp OR ovaries) AND (ct OR (computed AND tomography) OR (magnetic AND resonance) OR mri OR mr) AND (2004:py OR 2006:py OR 2007:py OR 2008:py OR 2009:py OR 2010:py OR 2011:py OR 2012:py OR 2013:py OR 2014:py OR 2015:py OR 2016:py OR 2017:py OR 2018:py OR 2019:py OR 2020:py OR 2021:py OR 2022:py OR 2023:py)

1.3 Web of Science search strategy

Available via apps.webofknowledge.com

Preliminary search date: 05 Jan 2023

Articles retrieved: 200

Formal search date: 06 Jan 2023

Articles retrieved: 200

#1 ((((TS=(textural)) OR TS=(texture)) OR TS=(radiomics)) OR TS=(radiomic)) OR TS=(machine learning)

#2 ((TS=(ovary)) OR TS=(ovarian)) OR TS=(ovaries)

#3 ((((TS=(computed tomography)) OR TS=(CT)) OR TS=(magnetic resonance)) OR TS=(MRI)) OR TS=(MR)

#4 #1 AND #2 AND #3

#5 #4 AND 2004 OR 2006 OR 2007 OR 2008 OR 2009 OR 2010 OR 2011 OR 2012 OR 2013 OR 2014 OR 2015 OR 2016 OR 2017 OR 2018 OR 2019 OR 2020 OR 2021 OR 2022 OR 2023(publication year)

((((TS=(textural)) OR TS=(texture)) OR TS=(radiomics)) OR TS=(radiomic)) OR TS=(machine learning) AND ((TS=(ovary)) OR TS=(ovarian)) OR TS=(ovaries) AND

((((TS=(computed tomography)) OR TS=(CT)) OR TS=(magnetic resonance)) OR TS=(MRI)) OR TS=(MR) AND 2004 OR 2006 OR 2007 OR 2008 OR 2009 OR 2010 OR 2011 OR 2012 OR 2013 OR 2014 OR 2015 OR 2016 OR 2017 OR 2018 OR 2019 OR 2020 OR 2021 OR 2022 OR 2023(publication year)

1.4 Cochrane Library search strategy

Available via www.cochranelibrary.com

Preliminary search date: 05 Jan 2023

Articles retrieved: 15

Formal search date: 06 Jan 2023

Articles retrieved: 15

#1 (ovary):ti,ab,kw OR (ovarian):ti,ab,kw OR (ovaries):ti,ab,kw

#2 (computed tomography):ti,ab,kw OR (CT):ti,ab,kw OR (magnetic resonance):ti,ab,kw OR (MRI):ti,ab,kw OR (MR):ti,ab,kw

#3 (textural):ti,ab,kw OR (texture):ti,ab,kw OR (radiomics):ti,ab,kw OR (radiomic):ti,ab,kw OR (machine learning):ti,ab,kw

#4 #1 AND #2 AND #3

((ovary):ti,ab,kw OR (ovarian):ti,ab,kw OR (ovaries):ti,ab,kw) AND ((computed tomography):ti,ab,kw OR (CT):ti,ab,kw OR (magnetic resonance):ti,ab,kw OR (MRI):ti,ab,kw OR (MR):ti,ab,kw) AND ((textural):ti,ab,kw OR (texture):ti,ab,kw OR (radiomics):ti,ab,kw OR (radiomic):ti,ab,kw OR (machine learning):ti,ab,kw)

1.5 Additional strategy

The search was expanded by screening the references of the retrieved articles for additional potentially eligible studies.

Articles retrieved: 17

This study search strategy has been tested in a pilot search to confirm its feasibility on 05 Jan 2023. The formal study search was performed on 06 Jan 2023.

2. Study selection criteria for systematic review and meta-analysis

2.1 Study selection criteria for systematic review

Inclusion criteria:

1) studies are reported in English with institutional full-text availability; 2) the cohort consists of patients with ovarian cancer; 3) the imaging modality are CT or MRI; 4) radiomics or texture features derived from medical images were used for analysis

Exclusion criteria:

1) duplicate studies; 2) case reports, conference abstracts, reviews or editorials; 3) studies concerning segmentation or feature processing algorithms only.

Contact with the authors was sought if the full-text version was not accessible otherwise.

2.2. Study selection criteria for meta-analysis

The studies included in meta-analysis should meet following criteria: 1) a radiomics model was constructed for analysis; 2) studies with clearly defined gold standard; 3) studies with documented two-by-two tables, sensitivity (Se), specificity (Sp), accuracy, positive likelihood ratio (PLR), negative likelihood ratio (NLR), and diagnostic odds ratio (DOR) with 95% confidence intervals (95% CIs), or with those could be calculated using published data.

Supplementary Study bibliographical information and model performance metrics

Supplementary Table S3 showed the characteristic of study population in each study. Patients with a large range of ages (10-92) were included, with various pathological types and in different treatment phase. The sample size of each study ranged from 28 to 1329.

The information about model development were summarized in Supplementary Table S4. Totally, MR images were applied in 35.1% (20/57) of the studies, while 64.9% (37/57) of them used CT images. The number of radiomics features extracted from imaging ranged from 3 to 9724 features. Seventeen and a half percent (10/57) of the studies contained features from metastatic tumors other than primary tumor of ovary, and 29.8% (17/57) of them extracted radiomics features from both primary and metastatic tumors. Almost all studies(55/57, 96.5%) implied feature selection methods. The most common used feature selection and modelling method was logistic regression.

Supplementary Table S1. PRISMA[1] diagnostic accuracy checklists

Section/topic	#	PRISMA-DTA Checklist Item	Reported on page #
TITLE / ABSTRACT			
Title	1	Identify the report as a systematic review (+/- meta-analysis) of diagnostic test accuracy (DTA) studies.	1
Abstract	2	Abstract: See PRISMA-DTA for abstracts.	2,3
INTRODUCTION			
Rationale	3	Describe the rationale for the review in the context of what is already known.	5
Clinical role of index test	D1	State the scientific and clinical background, including the intended use and clinical role of the index test, and if applicable, the rationale for minimally acceptable test accuracy (or minimum difference in accuracy for comparative design).	4,5
Objectives	4	Provide an explicit statement of question(s) being addressed in terms of participants, index test(s), and target condition(s).	5
METHODS			
Protocol and registration	5	Indicate if a review protocol exists, if and where it can be accessed (e.g., Web address), and, if available, provide registration information including registration number.	6
Eligibility criteria	6	Specify study characteristics (participants, setting, index test(s), reference standard(s), target condition(s), and study design) and report characteristics (e.g., years considered, language, publication status) used as criteria for eligibility, giving rationale.	6
Information sources	7	Describe all information sources (e.g., databases with dates of coverage, contact with study authors to identify additional studies) in the search and date last searched.	6
Search	8	Present full search strategies for all electronic databases and other sources searched, including any limits used, such that they could be repeated.	S2,3,4,5[2]

Study selection	9	State the process for selecting studies (i.e., screening, eligibility, included in systematic review, and, if applicable, included in the meta-analysis).	Fig.2
Data collection process	10	Describe method of data extraction from reports (e.g., piloted forms, independently, in duplicate) and any processes for obtaining and confirming data from investigators.	6
Definitions for data extraction	11	Provide definitions used in data extraction and classifications of target condition(s), index test(s), reference standard(s) and other characteristics (e.g. study design, clinical setting).	Table S2
Risk of bias and applicability	12	Describe methods used for assessing risk of bias in individual studies and concerns regarding the applicability to the review question.	6,7
Diagnostic accuracy measures	13	State the principal diagnostic accuracy measure(s) reported (e.g. sensitivity, specificity) and state the unit of assessment (e.g. per-patient, per-lesion).	8
Synthesis of results	14	Describe methods of handling data, combining results of studies and describing variability between studies. This could include, but is not limited to: a) handling of multiple definitions of target condition. b) handling of multiple thresholds of test positivity, c) handling multiple index test readers, d) handling of indeterminate test results, e) grouping and comparing tests, f) handling of different reference standards	8,9
Risk of bias across studies	15	Specify any assessment of risk of bias that may affect the cumulative evidence (e.g., publication bias, selective reporting within studies).	Page9& Figure S3
Additional analyses	16	Describe methods of additional analyses (e.g., sensitivity or subgroup analyses, meta-regression), if done, indicating which were pre-specified.	8,9
RESULTS			
Study selection	17	Provide numbers of studies screened, assessed for eligibility, included in the review (and included in meta-analysis, if applicable) with reasons for exclusions at each stage, ideally with a flow diagram.	Page9& Fig.2
Study characteristics	18	For each included study provide citations and present key characteristics including: a) participant characteristics (presentation, prior testing), b) clinical setting, c) study design, d) target condition	Table 1,TableS

		definition, e) index test, f) reference standard, g) sample size, h) funding sources	4&Table S5
Risk of bias and applicability	19	Present evaluation of risk of bias and concerns regarding applicability for each study.	Page11, Fig.3&TableS7
Results of individual studies	20	For each analysis in each study (e.g. unique combination of index test, reference standard, and positivity threshold) report 2x2 data (TP, FP, FN, TN) with estimates of diagnostic accuracy and confidence intervals, ideally with a forest or receiver operator characteristic (ROC) plot.	Fig.5&Figure S2
Synthesis of results	21	Describe test accuracy, including variability; if meta-analysis was done, include results and confidence intervals.	12
Additional analysis	23	Give results of additional analyses, if done (e.g., sensitivity or subgroup analyses, meta-regression; analysis of index test: failure rates, proportion of inconclusive results, adverse events).	11,12,13
DISCUSSION			
Summary of evidence	24	Summarize the main findings including the strength of evidence.	13,14,15,16
Limitations	25	Discuss limitations from included studies (e.g. risk of bias and concerns regarding applicability) and from the review process (e.g. incomplete retrieval of identified research).	16,17
Conclusions	26	Provide a general interpretation of the results in the context of other evidence. Discuss implications for future research and clinical practice (e.g. the intended use and clinical role of the index test).	17
FUNDING			
Funding	27	For the systematic review, describe the sources of funding and other support and the role of the funders.	13

[1] *PRISMA: Preferred Reporting Items for Systematic Reviews and Meta-analyses Source: McInnes et al [18].*

[2] *S means Supplementary Materials*

Supplementary Table S2 Data collection instrument definitions

Field	Item	Explanation
Bibliographical information	Title	The title of the study
	Authors	The authors of the study
	Author specialty	The specialty of the first author
	Year	Published year
	Country	Geographical origin of the study
	Journal	Published journal
	Journal speciality	The main topic of the journal
	Volume	Published volume
	Issue	Published issue
	Page	Published page
	If	The impact factor of the journal
	Study id	Internal Study ID, determined by First author + Year + Journal, if needed
Baseline study information	Study design	Prospective or retrospctive
	Research question	The aim of the study
	Outcome	The outcome of the study
	Age	The mean/median age of study population

Participant characteristics	Detailed characteristics of patients included in the study
Pathological types	The pathological types of ovarian cancers in patients
Figo stage	The FIGO stage of ovarian cancers in patients
Reference standard	Standards for practical application to define the dependent variable
Interval	Interval between the application of imaging and reference standard
Negative result	Was a negative result published in the article?
Imaging technique	CT or MR
Mri sequence	T1, T2, ADC, DWI...
Mri manufacturer	Siemens, GE, Philips…
Mri model	Avanto, Symphony…
Mri echo time	Echo time
Mri repetition time	Repetition time
Mrie train length	Echo train length
Mrib values	The b-values for calculation of adc map
Mri contrast phase	Name of contrast phase (i.e. Portal venous)
Mri contrast timing	Seconds after injection
Mri flip angle	Flip angle
Mri thickness	Slide thickness (mm)
Mri respiration	Respiratory triggered, breath hold
Ct enhancement	Unenhanced, enhanced or both

	Ct manufacturer	Siemens, GE, Philips…
	Ct model	SOMATOM Force, Aquilon ONE…
	Ct kernel	Vendor specific (B31s) or general (soft, bone)
	Ct tube voltage	Tube voltage
	Ct tube current	Tube current (auto, 250mAs)
	Ct contrast phase name	Arterial, portal venous…
	Ct timing	Bolus tracking, 25s…
	Ct slice thickness	Slice thickness (mm)
Modeling information	Segmentation software	Software used for segmentation
	Preprocess	Were the imaging preprocessed before feature extraction?
	Initial feature type	The types of extracted radiomics features
	Final feature type	The types of radiomics features after selection
	Extraction software	Software used for feature extraction
	Selection method	The method used for radiomicsfeature selection
	Modeling method	The method used for building a radiomics model
Model performance metrics	Data type	Was the model constructed base on only radiomics data or radiomics data combined with non-texture measurements?
	Datasets type	Was the model constructed based on the training or testing dataset?
	P	Number of patients in positive condition
	N	Number of patients in negative condition

	Tp	True positive
	Tn	True negative
	Fn	False positive
	Tn	False negative
	Sen	Sensitivity
	Spe	Specificity
	Acc	Accuracy
	Auc	Area under curve
Elements of the RQS	Protocol clear	Is the imaging protocol reproducible?
	Registration	Registration of multiple phases (yes, no)
	Segmentation multiple	Has segmentation been performed by multiple readers (yes/no)
	Segmentation technique	manual, semi-automatic, automatic
	Segmentation description	2D or 3D segmentation
	Phantom	Has a phantom scan been performed (yes/no)
	Phantom kind	What kind of phantom was used?
	Phantom parameters	Were the scanning parameters with the phantom identical to the patient parameters?
	Nr scanners	How many scanners were used to obtain the data?
	Multiple timepoints	Was repeat imaging carried out for texture comparison?
	Multiple	Were multiple tests conducted?

Multiple Method p Value	Were p Values adjusted for multiple testing?
Multiple method feature reduction	Was the number of features reduced to reduce the risk of false positives due to multiple testing?
Multiple nr comp features	Number of features collected in total
Nr datasets	Number of Patients in the study
Multiple nr selelected features	Number of features input in final model
Multivariate	Was a multivariate analysis performed?
Multivariate outcomes	Is the multivariate analysis used for the perdition of the primary outcome?
Multivariate predictive power	Is the predictive power of the multivariate analysis reported?
Multivariate non radiomics	Did the multivariate analysis include non-texture measurements (e.g. tumor volume)?
Biological correlates	Was the model correlated to a biological endpoint (e.g. VHL mutation, VEGF expression)?
Cut off	Was a cut off analysis performed?
Cut off prior	Was the cut off selected prior to the analysis?
Cut off method	Where was the cut off selected (median, best discrimination)?
Discrimination	What discrimination statistic was used (AUC, Sensitivity/Specificity, none…)
Calibration	Was any kind of calibration statistic used?
Prospective plan	Was the study planned prospectively?

	Prospective data	Was the study data acquired prospectively?
	Retrospective data	Was the data acquired retrospectively?
	Validation	Was a validation cohort used?
	Validation internal	Was an internal (=same hospital) validation cohort used?
	Validation external	Was an external (=other hospital) validation cohort used?
	Validation external nr cohorts	How many external validation cohorts were used?
	Validation parameters constant	Was the prediction model kept constant for the validation cohort?
	Nr centers	How many centers does the data come from?
	Gold standard	Was the gold standard used to define the dependent variable (e.g. Histology for tumor type)?
	Gold standard comparison	Is the model the same as/superior to the current “gold standard”?
	Clinical applications	Does the article mention clinical applications of texture analysis?
	Clinical applications relevance	Are these clinically relevant applications?
	Cost-effectiveness	Was a cost- effectiveness analysis performed?
	Open science	Were images, segmentations, code made available publicly?
QUADAS-2 tool tailored to our research question	Sample type	Was a consecutive or random sample of patients enrolled??
	Study design	Was a case-control design avoided?
	Exclusion	Did the study avoid inappropriate exclusions?
	Selection applicability	Is there concern that the included patients do not match the review question?

	Independent index test	Were the index test results interpreted without knowledge of the results of the reference standard?
	Threshold	If a threshold was used, was it pre-specified?
	Index test applicability	Is there concern that the index test, its conduct, or interpretation differ from the review question?
	Reference clear	Is the reference standard likely to correctly classify the target condition?
	Independent reference	Were the reference standard results interpreted without knowledge of the results of the index test?
	Standard applicability	Is there concern that the target condition as defined by the reference standard does not match the review question?
	Appropriate interval	Was there an appropriate interval between index test(s) and reference standard?
	Whole patient	Were all patients included in the analysis?
-	Primary endpoint	What was the primary endpoint of the study?

Note: QUADAS-2, quality assessment diagnostic accuracy studies 2; RQS, radiomics quality score, Nr, number

Supplementary Table S3. RQS checklist

No.	RQS scoring item	Points and Interpretation
1	**Image protocol quality -** well-documented image protocols (for example, contrast, slice thickness, energy, etc.) and/or usage of public image protocols allow reproducibility/replicability	+ 1 if protocols are well-documented<br>+ 1 if public protocol is used
2	**Multiple segmentations -** possible actions are: segmentation by different physicians/algorithms/software, perturbing segmentations by (random) noise, segmentation at different breathing cycles. Analyse feature robustness to segmentation variabilities	+ 1 if segmented multiple times (different physicians, algorithms, or perturbation of regions of interest)
3	**Phantom study on all scanners -** detect inter-scanner differences and vendor-dependent features. Analyse feature robustness to these sources of variability	+ 1 if texture phantoms were used for feature robustness assessment
4	**Imaging at multiple time points -** collect images of individuals at additional time points. Analyse feature robustness to temporal variabilities (for example, organ movement, organ expansion/ shrinkage)	+ 1 multiple time points for feature robustness assessment
5	**Feature reduction or adjustment for multiple testing -** decreases the risk of overfitting. Overfitting is inevitable if the number of features exceeds the number of samples. Consider feature robustness when selecting features	- 3 if neither measure is implemented<br>+ 3 if either measure is implemented

6	**Multivariable analysis with non-radiomics features** (for example, EGFR mutation) - is expected to provide a more holistic model. Permits correlating/inferencing between radiomics and non-radiomics features	+ 1 if multivariable analysis with non-radiomics features
7	**Detect and discuss biological correlates** - demonstration of phenotypic differences (possibly associated with underlying gene–protein expression patterns) deepens understanding of radiomics and biology	+ 1 if present
8	**Cut-off analyses** - determine risk groups by either the median, a previously published cut-off or report a continuous risk variable. Reduces the risk of reporting overly optimistic results	+ 1 if cutoff either pre-defined or at median or continuous risk variable reported
9	**Discrimination statistics** - report discrimination statistics (for example, C-statistic, ROC curve, AUC) and their statistical significance (for example, p-values, confidence intervals). One can also apply resampling method (for example, bootstrapping, cross-validation)	+ 1 if a discrimination statistic and its statistical significance are reported<br>+ 1 if a resampling method technique is also applied
10	**Calibration statistics** - report calibration statistics (for example, Calibration-in-the-large/slope, calibration plots) and their statistical significance (for example, *P*-values, confidence intervals). One can also apply resampling method (for example, bootstrapping, cross-validation)	+ 1 if a calibration statistic and its statistical significance are reported<br>+ 1 if a resampling method technique is also applied
11	**Prospective study registered in a trial database** - provides the highest level of evidence supporting the clinical validity and usefulness of the radiomics biomarker	+ 7 for prospective validation of a radiomics signature in an appropriate trial
12	**Validation** - the validation is performed without retraining and without adaptation of the cut-off value, provides crucial information with regard to credible clinical performance	− 5 if validation is missing<br>+ 2 if validation is based on a dataset from the same institute/<br>+ 3 if validation is based on a dataset from another institute/<br>+ 4 if validation is based on two datasets from two distinct institutes/

		+4 if the study validates a previously published signature/<br>+5 if validation is based on three or more datasets from distinct institutes<br>*Datasets should be of comparable size and should have at least 10 events per model feature
13	**Comparison to ‘gold standard**’ - assess the extent to which the model agrees with/is superior to the current ‘gold standard’ method (for example, TNM-staging for survival prediction). This comparison shows the added value of radiomics	+ 2 for comparison to gold standard
14	**Potential clinical utility** - report on the current and potential application of the model in a clinical setting (for example, decision curve analysis)	+ 2 for reporting potential clinical utility
15	**Cost-effectiveness analysis** - report on the cost-effectiveness of the clinical application (for example, QALYs generated)	+ 1 for cost-effectiveness analysis
16	**Open science and data** - make code and data publicly available. Open science facilitates knowledge transfer and reproducibility of the study	+ 1 if scans are open source<br>+ 1 if region of interest segmentations are open source<br>+ 1 if code is open source<br>+ 1 if radiomics features are calculated on a set of representative ROIs and the calculated features and representative ROIs are open source
Total points (36 = 100%)		

Note: RQS, radiomics quality score

Supplementary Table S4. Characteristics of study population in each study

No.	StudyID	Ref	Participant characteristics	Age in years(range)	Pathological types	Sample Size
1	Zheng2022	(1)	untreated, histopathologically confirmed, BEOT or MEOT, preoperative MRI	D: SBOT:59.38(NR), SMOT:57.55(NR); IV: SBOT:62.06(NR), SMOT:54.93(NR)	86 SBOT/70 SMOT	156
**2**	**Zhang2022**	**(2)**	**untreated, histopathologically confirmed, EOT or MOT, primary tumor for MOT invisible on CT**	**D: 54.25(NR) IV: 54.43(NR)**	**201 EOT/85 MOTs**	**286**
**3**	**Xu2022**	**(3)**	**untreated, histopathologically confirmed, BEOT or EOC, preoperative MRI within 1 month**	**BEOT: 38.5(NR), EOC: 57.0(NR); Type I EOC: 55.0(NR), Type II EOC: 58.0(NR)**	**34 BEOT/112 EOC**	**146**
**4**	**Wei2022**	**(4)**	**untreated, histopathologically confirmed, Benign or borderline EOT, preoperative MRI within 2 weeks**	**D: 45.49(NR) IV: 46.94(NR) EV: 44.70(NR)**	**209 serous/180 mucinous/28 others**	**417**
5	M.Wang2022	(5)	untreated, histopathologically confirmed, HGSC or non-HGSC of EOC, preoperative ceCT	53.6(18-90)	436HGSC/229 non-HGSC	665
**6**	**Nagawa2022**	**(6)**	**untreated, histopathologically confirmed, OTFGs OR OGCTs, preoperative MRI within 2 months**	**OTFG: 59.3(NR) OGCT: 57.5(NR)**	**32OTFG/21OGCT**	**53**

No.	StudyID	Ref	Participant characteristics	Age in years(range)	Pathological types	Sample Size
**7**	**LiuX2022**	**(7)**	**untreated, histopathologically confirmed, BEOT or MEOT, preoperative MRI**	**BEOT: 39.8(NR); MEOT: 51.9(NR)**	**91 OBT/83 HGSC/7 mucinous carcinomas/4 mixed carcinomas/5 CCC/3 EC/3 LGSC**	**196**
**8**	**LiuP2022**	**(8)**	**histopathologically confirmed, benign or malignant ovarian tumor**	**47(10-79)[a]**	**73benign/62malignant**	**135**
**9**	**LiS2022**	**(9)**	**untreated, histopathologically confirmed, ovarian tumor, preoperative pelvic CT within half a months**	**NR**	**64benign/76malignant**	**140**
**10**	**LiJ.1.2022**	**(10)**	**untreated, histopathologically confirmed, ovarian tumor, pretreatment contrast-enhanced CT**	**D: 51.00(NR) IV: 50.00(NR)[a]**	**719benign/610malignant**	**1329**
11	LiJ.2.2022	(11)	untreated, histopathologically confirmed, EOC, pretreatment contrast-enhanced CT	D: 54(NR) IV: 56(NR)[a]	162Type I/308 Type II	470
**12**	**Zhu2021**	**(12)**	**untreated, histopathologically confirmed OC, preoperative neCT up to 1 month before surgery**	**54.23(15-79)**	**86EOC/15NEOC**	**101**
**13**	**YuXP2021**	**(13)**	**untreated, histopathologically confirmed, early-stage SBOT or SMOT**	**D: SBOT:41.2(NR), SMOT:52.5(NR); IV: SBOT:40.2(NR), SMOT:54.5(NR)**	**80 SBOT/102 SMOTs.**	**182**

No.	StudyID	Ref	Participant characteristics	Age in years(range)	Pathological types	Sample Size
14	Ye2021	(14)	untreated, histopathologically confirmed, BEOT or FIGO stage I/II MEOT, preoperative MRI	BEOT:46(28.0-60.3) MEOT:52(46.0-57.3)	36 BEOTs/70 MEOTs	88
15	Song.1.2021	(15)	untreated, ultrasound suggested ovarian masses with solid components	Benign:45.07(NR), Borderline: 44.56(NR), Malignant:51.61(NR)	33 benign/18 borderline/53 malignant	82
16	Park2021	(16)	incidental ovarian lesion with known outcome, CT images available	Benign: 44(19-87); Malignant: 61(20-92)	60 Benig/79 Malignant	427
**17**	**LiS2021**	**(17)**	**untreated, histopathologically confirmed ovarian tumor, preoperative CT within half a month**	**D: benign:41.6(NR), maglignant:53.4(NR); IV: benign:41.3(NR), maglignant:52.8(NR)**	**NR**	**160**
**18**	**LiN2021**	**(18)**	**untreated, histopathologically confirmed OGCT or OTCA-FTCA, MRI images available**	**OGCT: 52.93(15-71) OTCA-FTCA: 49.93(24-94)**	**NR**	**46**
19	Jian2021	(19)	untreated, histopathologically confirmed EOC, preoperative MRI within a month	D: 53.51(NR); IV: 51.24(NR); EV: 57.21(NR)	19 LGSC/31 Mucinous/29EC/64 CCC/150 HGSC/1 Undifferentiated	294
**20**	**Hu2021**	**(20)**	**patients with POC or SOC**	**D: POC:54.74 (NR), SOC:50.79 (NR) IV: POC:58.21(NR), SOC:53.53(NR)**	**62 POC 48 SOC:20 gastric cancer/15 colorectal cancer/ 13 sigmoid colon cancer**	**110**

No.	StudyID	Ref	Participant characteristics	Age in years(range)	Pathological types	Sample Size
21	An2021	(21)	untreated, histopathologically confirmed EOC, preoperative ceCT	52(NR)	133 HGSC/72 non-HGSC	205
**22**	**Qian2020**	**(22)**	**untreated, histopathologically confirmed EOC, preoperative MRI**	**51.8(29-81)**	**24 type/37 typeII**	**61**
**23**	**Lupean2020**	**(23)**	**histopathologically confirmed, benign or malignant ovarian cysts**	**39.28(24-78)**	**15 benign/13 malignant**	**28**
24	Li2020	(24)	untreated, histopathologically confirmed BEOT or MEOT, preoperative MRI within 2 weeks	D:47.20(NR); IV:48.98(NR); EV:51.62(NR)	165 BEOT/336 MEOT	501
**25**	**Zhang2019**	**(25)**	**patients with clinically suspected gynecological diseases, preoperative MRI**	**52.7(NR)**	**97 Type I/80 Type II; 28 Others malignancies/75 Benign etiologies**	**280**
26	Rundo2022	(26)	Aged 18 years or older, histopathologically confirmed as HGSOC, NACT before DPS, pre- and post-NACT contrast-enhanced CT of the abdomen and pelvis	D: non-response: 63(NR) response: 62(NR) IV: non-response: 62(NR) response: 64(NR)	109HGSOC	109
27	Zargari2018	(27)	recurrent ovarian/peritoneal/tubal carcinoma of high grade histology, treated with systemic chemotherapy after the primary cytoreduction, pre- and post-therapy CT	responders: 66(NR) non-responders: 69(NR)	Recurrent, high grade (serous, endometrioid, undifferentiated) ovarian/peritoneal/tubal carcinoma	120

No.	StudyID	Ref	Participant characteristics	Age in years(range)	Pathological types	Sample Size
28	Danala2017	(28)	recurrent ovarian/peritoneal/tubal carcinoma of high grade histology, treated with systemic chemotherapy, pre- and post-therapy CT	responders: 66(NR) non-responders: 67(NR)	Recurrent, high grade (serous, endometrioid, undifferentiated) ovarian/peritoneal/tubal carcinoma	91
29	Qiu2016	(29)	recurrent ovarian/peritoneal/tubal carcinoma of high grade histology, pre- and post-therapy CT	67.9(50-84)	High grade (serous, endometrioid, undifferentiated) ovarian/peritoneal/tubal carcinoma	30
**30**	**Wan2023**	**(30)**	**untreated, histopathologically confirmed, OC, pretreatment CT, from TCGA or TCIA**	**NR**	**NR**	**146**
**31**	**Wu2022**	**(31)**	**histopathologically confirmed as HGSOC, treated with cytoreductive surgery and 6–8 cycles of platinum-based chemotherapy, aged 18-70**	**53.65(NR)**	**110HGSOC**	**110**
32	WangT2022	(32)	untreated, histopathologically confirmed EOC, preoperative MRI	47.7 (NR)	55 BEOT/23 CCC/12 EC/9 LGSC/87 HGSC	186
**33**	**Lu2022**	**(33)**	**histopathologically confirmed HGSOC, preoperative pelvic MRI followed by primary debulking surgery**	**57(37-79)**	**128 advanced HGSOC**	**128**

No.	StudyID	Ref	Participant characteristics	Age in years(range)	Pathological types	Sample Size
**34**	**LiC2022**	**(34)**	**histopathologically confirmed HGSOC, preoperative pelvic MRI followed operation, with at least 18-months follow-up**	**54(41-78)[a]**	**141HGSOC**	**141**
**35**	**Hu2022**	**(35)**	**histopathologically confirmed HGSOC, CT within two months before surgery, availability of OS and DFS data**	**D: 50(20-73),<br>IV: 51.5(18-73),<br>EV: 55(32-68)**	**217HGSOC**	**217**
36	Hong2022	(36)	histopathologically confirmed serous ovarian cancer, contrast-enhanced abdomen and pelvic CT performed before surgery	D: 59(NR)<br>EV: 57(NR)	119SOC	119
37	Gao2022	(37)	histopathologically confirmed ovarian cancer, pretreatment CT	NR	NR	343
38	Fotopoulou2022	(38)	histopathologically confirmed, HGSOC, pretreatment portal venous CT	NR	547HGSOC	547
39	Feng2022	(39)	contrast-enhanced CT images from TCIA	NR	NR	59
40	Boehm2022	(40)	histopathologically confirmed, HGSOC, pre-treatment contrast-enhanced abdominal/pelvic CT	NR	338HGSOC	338
41	Avesani2022	(41)	histopathologically confirmed HGSOC, pretreatment contrast-enhanced CT study of at least	Group1: 53(36-76)<br>Group2: 58(41-81)<br>Group3: 63(29-86)	218HGSOC	218

No.	StudyID	Ref	Participant characteristics	Age in years(range)	Pathological types	Sample Size
			abdomen and pelvis in portal-venous phase followed by staging or complete debulking, availability of BRCA1-2 mutational status	Group4: 58(31-83)		
**42**	**YuXY2021**	**(42)**	**untreated, histopathologically confirmed EOC, preoperative MRI within one week**	**54(33-82)**	**28 TypeI/58 TypeII**	**86**
43	Yi2021	(43)	histopathologically confirmed stage II-IV EOC, standard platinum-based chemotherapy within 7 days after maximal cyto-reductive surgery	52.2(NR)	Serous or mucinous epithelial OC	102
**44**	**Song.2.2021**	**(44)**	**patients with untreated primary malignant ovarian tumors, preoperative MRI within 20 days**	**51.944(NR)**	**NR**	**89**
**45**	**Liu2021**	**(45)**	**untreated, histopathologically confirmed EOC, preoperative ceCT**	**BRCA non-mutation: 54.86(35-75) BRCA mutation: 53.93(36-77)**	**NR**	**106**
**46**	**LiM2021**	**(46)**	**histopathologically confirmed advanced EOC, with NGS gene test, ceCT performed preoperatively or during chemoradiotherapy.**	**57(36-79)**	**63 serous/13 mucinous/15 CCC/4 EC**	**95**
47	LiH.1.2021	(47)	histopathologically confirmed advanced HGSOC, platinum-based	55(36-76)	advanced HGSOC	117

No.	StudyID	Ref	Participant characteristics	Age in years(range)	Pathological types	Sample Size
			chemotherapy after primary debulking surgery			
48	LiH.2.2021	(48)	histopathologically confirmed advanced HGSOC, preoperative MRI, appropriate surgical staging and debulking surgery followed by systemic chemotherapy	56(36-79)	advanced HGSOC	217
**49**	**Chen.1.2021**	**(49)**	**histopathologically confirmed HGSOC, preoperative ceCT**	**Mean: D: Recurrence free: 52.59(NR), Recurrence: 50.38(NR) IV: Recurrence free: 52.98(NR), Recurrence: 50.63(NR)**	**HGSOC**	**256**
**50**	**Chen.2.2021**	**(50)**	**untreated, histopathologically confirmed HGSOC, preoperative ceCT within 2 weeks**	**D: negative: 53.69(NR), positive: 50.32(NR) IV: negative: 54.37(NR), positive: 48.31(NR)**	**HGSOC**	**256**
**51**	**Ai2021**	**(51)**	**OC patients with primary debulking surgery, preoperative neCT within 1 month**	**54.23(15-79)[a]**	**86 EOC/15 NEOC**	**101**

No.	StudyID	Ref	Participant characteristics	Age in years(range)	Pathological types	Sample Size
**52**	**Veeraraghavan2020**	**(52)**	**stage III-IV HGSOC patients, preoperative ceCT, attempted primary cytoreduction, at least two disease sites, and molecular analysis performed within TCGA**	**MSKCC: 59(NR), TCIA: 61(NR)[a]**	**HGSOC**	**75(40+35)**
53	Wei2019	(53)	histopathologically confirmed advanced HGSOC, preoperative ceCT followed by primary debulking surgery	50(NR)[a]	HGSOC	142(100+42)
54	Meier2019	(54)	histopathologically confirmed advanced HGSOC, preoperative ceCT followed by primary debulking surgery	75(32-82)[a]	HGSOC	88
55	Lu2019	(55)	histopathologically confirmed EOC	HH cohort: 62(19-91) TCGA cohort:59(40-81)[a]	231 serous/58 non-serous/5 unknown; 16 low grade/254 high grade/24 unknown	364
56	Rizzo2018	(56)	histopathologically confirmed HGSOC, pretreatment CT followed by cytoreductive surgery, availability of BRCA1-2 mutational status	53(36-76)[a]	HGSC	101
57	Vargas2017	(57)	histopathologically confirmed FIGO stage IIIC-IV HGSOC with at least three tumor sites, preoperative	NR	HGSOC	38

No.	StudyID	Ref	Participant characteristics	Age in years(range)	Pathological types	Sample Size
			standard of care CT followed by primary cytoreductive surgery			

Note: Bold studies included in the meta-analysis. Age in years(range) were reported by mean, with the [a] for median.

Abbreviation: BEOT: borderline epithelial ovarian tumor, MEOT: malignant epithelial ovarian tumor, EOC: epithelial ovarian cancer, NEOC: non-epithelial ovarian cancer, SBOT: serous borderline ovarian tumor, SMOT: serous malignant ovarian tumor, OGCT: ovarian granulosa cell tumor, OTCA–FTCA: thecoma-fibrothecoma, POC: primary ovarian cancer, SOC: secondary ovarian cancer, HGSC: high grade serous carcinoma, HGSOC: high-grade serous ovarian carcinoma, CCC: clear cell type carcinomas, EC: endometrioid carcinomas, OC: ovarian cancer, D: development, IV: internal validation, EV external validation, NR: not reported.

Supplementary Table S5. Detailed radiomics model characteristics in each study

No.	StudyID	Modality	Segmentation	No of Reviewer	ROI	Segmentation software	No of features extracted	Types of Radiomics feature extracted	Feature extraction software	Features from sites other than ovaries	Features from multiple sites	Feature selection method	Modeling method
1	Zheng2022	3.0T MRI	Manual	Two	3D	3D-slicer	1612	Shape, first order, GLCM, GLRLM, GLSZM, GLDM, wavelet.	Pyradiomics	No	Yes	LassoCV	SVM, RF, LR, XGB
**2**	**Zhang2022**	**CT**	**Manual**	**Two**	**3D**	**ITK-SNAP**	**1120**	**Size, shape, first order, second order(GLCM, GLRLM, GLZSM)**	**Analysis Kit**	**No**	**No**	**Spearman rank correlation test, mRMR, LASSO**	**LASSO**
**3**	**Xu2022**	**3.0T MRI**	**Manual**	**Single**	**2D**	**ITK-SNAP**	**390**	**Shape, first order, high order**	**Pyradiomics**	**No**	**Yes**	**reproducibility, Wilcoxon rank-sum test, LASSO, multivariate LR**	**stepwise multivariate LR**
**4**	**Wei2022**	**1.5T or 3.0T MRI**	**Manual**	**Two**	**3D**	**ITK-SNAP**	**1130**	**Original, wavelet**	**Pyradiomics**	**No**	**No**	**ICC analysis, Mann-Whitney U test, RF,**	**LR, SVM, RF, NB**

No.	StudyID	Modality	Segmentation	No of Reviewer	ROI	Segmentation software	No of features extracted	Types of Radiomics feature extracted	Feature extraction software	Features from sites other than ovaries	Features from multiple sites	Feature selection method	Modeling method
												**Spearman correlation analysis, LASSO**	
5	M.Wang 2022	CT	Manual	Two	3D	3D-slicer or ITK-SNAP	1288	shape, first order, GLCM, GLSZM, GLRLM, GLDM, NGTDM, LoG, wavelet	Pyradiomics	No	No	Mann-Whitney U test, LASSO	LR
**6**	**Nagawa 2022**	**3.0T MRI**	**Manual**	**Two**	**2D**	**ITK-SNAP**	**88**	**First order, GLCM, GLRLM, GLSZM, 14 GLDM, NGTDM.**	**Pyradiomics**	**No**	**No**	**LASSO**	**Tenfold cross-validation**
**7**	**LiuX2022**	**1.5T MRI**	**Manual**	**Single**	**2D& 3D**	**ITK-SNAP**	**396**	**NR**	**Analysis Kit**	**No**	**No**	**LASSO**	**LASSO**
**8**	**LiuP2022**	**CT**	**Manual**	**Single**	**3D**	**ITK-SNAP**	**9724**	**Nontexture, Global, GLCM, GLRLM, GLSZM, NGTDM**	**Toolkit by Vallieres et al.**	**No**	**NR**	**Mann-whitney u test, the Pearson correlation**	**SVM**

No.	StudyID	Modality	Segmentation	No of Reviewer	ROI	Segmentation software	No of features extracted	Types of Radiomics feature extracted	Feature extraction software	Features from sites other than ovaries	Features from multiple sites	Feature selection method	Modeling method
												coefficient, elastic net(linear combination of LASSO and ridge regression), RFECV	
9	LiS2022	CT	Manual	Two	2D& 3D	ITK-SNAP	396	texture, histogram, shape, GLCM, GLRLM, GLSZM, GLCM, RLM	Artificial intelligence life science toolkit	No	No	Spearman rank correlation test, mRMR, LASSO	LR
10	LiJ.1.2022	CT	Manual	Two	3D	3D-slicer	1316	Shape, first order, second order(GLCM, GLRLM, GLSZM, GLDM, NGTDM),	Pyradiomics	No	No	Univariate analysis, Pearson or Spearman correlation matrices, RF	KNN, SVM, RF, LR, MLP, XGBoost

No.	StudyID	Modality	Segmentation	No of Reviewer	ROI	Segmentation software	No of features extracted	Types of Radiomics feature extracted	Feature extraction software	Features from sites other than ovaries	Features from multiple sites	Feature selection method	Modeling method
								**LoG features, wavelet.**					
11	LiJ.2.2022	CT	Manual	Two	3D	3D-slicer	1319	Shape, first order, GLCM, GLRLM, GLSZM, GLDM, NGTDM, wavelet, LoG	Pyradiomics	No	No	ICC, univariate analysis, LASSO	SVM, KNN, NB, LR, XGBoost, RF
**12**	**Zhu2021**	**CT**	**Manual**	**Single**	**3D**	**LIFEx**	**148**	**First order (histogram, shape, conventional statistics), second order (GLCM, NGLDM, GLRLM, GLZLM)**	**LIFEx**	**No**	**No**	**Mann-Whitney U-test, LASSO**	**LR**
**13**	**YuXP2021**	**CT**	**Manual**	**Two**	**3D**	**3D-slicer**	**1167**	**Shape, first order, GLCM, GLRLM, GLSZM, NGTDM, GLDM.**	**3D-slicer**	**No**	**No**	**ICC, LASSO**	**SVM**

No.	StudyID	Modality	Segmentation	No of Reviewer	ROI	Segmentation software	No of features extracted	Types of Radiomics feature extracted	Feature extraction software	Features from sites other than ovaries	Features from multiple sites	Feature selection method	Modeling method
14	Ye2021	3T MRI	Manual	Single	3D	MaZda	314	Texture (Histogram, Absolute gradient, RLM, GLCM)	MaZda	No	Yes	Fisher, POE+ACC, MI, RF	LR
15	Song.1.2021	3T MRI	Manual	Single	3D	ITK-SNAP	6720	Shape, first order, GLCM, GLRLM, GLSZM, GLDM	PyRadiomics	No	Yes	2-class classification task: ICC, Mann-Whitney U-test，PCC，multivariate LR 3-class classification task: ICC, a 10-fold cross-validated recursive feature elimination	LR

No.	StudyID	Modality	Segmentation	No of Reviewer	ROI	Segmentation software	No of features extracted	Types of Radiomics feature extracted	Feature extraction software	Features from sites other than ovaries	Features from multiple sites	Feature selection method	Modeling method
												based on tree bagging	
16	Park2021	CT	Manual	Single	2D	TexRAD	NR	Total pixel, mean, SD, entropy, MPP, skewness, kurtosis, entropy	TexRAD	No	No	Wilcoxon rank-sum test,Pearson correlations	SVM, RF, LR
**17**	**LiS2021**	**CT**	**Manual**	**Two**	**2D**	**ITK-SNAP**	**396**	**Texture, histograms, form factors, GLCM, GLRLM, GLZSM**	**Artificial Intelligence Kit**	**No**	**No**	**Spearman rank correlation test,mRMR,LASSO**	**LR**
**18**	**LiN2021**	**3T MRI**	**Manual**	**Two**	**2D**	**ITK-SNAP**	**1316**	**Mean, entropy, energy, skewness, kurtosis, and standard deviation**	**Analysis Kit**	**No**	**No**	**Mann–Whitney U-test，univariate LR，mRMR，multivariate LR**	**LR**

No.	StudyID	Modality	Segmentation	No of Reviewer	ROI	Segmentation software	No of features extracted	Types of Radiomics feature extracted	Feature extraction software	Features from sites other than ovaries	Features from multiple sites	Feature selection method	Modeling method
19	Jian2021	1.5T/3T MRI	Manual	Single	3D	MITK, ITK	851	Shape, first order, GLCM, GLRLM, GLSZM, NGTDM, GLDM, wavelet	NR	No	No	Pearson correlation matrixes，mRMR	LASSO
**20**	**Hu2021**	**CT**	**Manual**	**Single**	**3D**	**ITK-SNAP**	**396**	**Histogram, formfactor, GLSZM, RLM**	**Artificial Intelligence Kit**	**No**	**No**	**ANOVA + KW test, binary LR, LASSO Cox LR，Multivariate LR**	**LR**
21	An2021	CT	Manual	Two	2D	TexRAD	36	Mean grey-level intensity, SD, entropy, MPP, skewness, kurtosis	TexRAD	No	No	ICC, univariate ROC curve analysis, excluding mutual correlations	RF

No.	StudyID	Modality	Segmentation	No of Reviewer	ROI	Segmentation software	No of features extracted	Types of Radiomics feature extracted	Feature extraction software	Features from sites other than ovaries	Features from multiple sites	Feature selection method	Modeling method
**22**	**Qian2020**	**3T MRI**	**Manual**	**Two**	**3D**	**NR**	**1070*4**	**Shape, first order, GLCM, GLSZM, GLRLM, GLDM**	**PyRadiomics**	**No**	**Yes**	**ANOVA, Kruskal-Wallis test, univariate LR, LASSO**	**LR**
**23**	**Lupean 2020**	**1.5T MRI**	**Manual**	**Single**	**2D**	**TexRAD**	**24**	**Mean, SD, MPP, skewness, kurtosis**	**TexRAD**	**No**	**No**	**univariate, multivariate, ROC analyses**	**NR**
24	Li2020	1.5T/3T MRI	Manual	Two	3D	MITK	851	Shape, first order, GLCM , GLSZM, GLRLM, NGTDM, GLDM, wavelet	Pyradiomics	No	No	ICC, PCC, mRMR	LASSO, multivariable LR
**25**	**Zhang2019**	**1.5T MRI**	**Manual**	**Single**	**2D**	**MATLAB**	**1714**	**Intensity, shape, texture, wavelets**	**NR**	**No**	**No**	**Differentiating: ISR Survival analysis: Kaplan-Meier plot, LASSO**	**Differentiating: SVM; survival analysis: LASSO**

No.	StudyID	Modality	Segmentation	No of Reviewer	ROI	Segmentation software	No of features extracted	Types of Radiomics feature extracted	Feature extraction software	Features from sites other than ovaries	Features from multiple sites	Feature selection method	Modeling method
													regression
26	Rundo2022	CT	Manual	Multiple	3D	Microsoft Radiomics App	107	First order, GLCM, GLRLM, GLSZM, GLDM, NGTDM	Pyradiomics	Yes	Yes	Spearman's correlation	Elastic Net LR
27	Zargari2018	CT	Semi-automatic	Single	3D	CAD scheme	133	Shape & density, GLDM, FFT, DCT, wavelet	CAD scheme	Yes	Yes	ROC analysis, PSO algorithm	GLM
28	Danala2017	CT	Semi-automatic	NR	3D	CAD scheme	159	Density, shape, texture, and wavelets	CAD scheme	Yes	Yes	ROC analysis, nearest neighbor error algorithm	Equal-weighted fusion models
29	Qiu2016	CT	Semi-automatic	NR	3D	CAD scheme	3	Tumor volume, density, SD	CAD scheme	Yes	Yes	ROC analysis,	Linear combinat

No.	StudyID	Modality	Segmentation	No of Reviewer	ROI	Segmentation software	No of features extracted	Types of Radiomics feature extracted	Feature extraction software	Features from sites other than ovaries	Features from multiple sites	Feature selection method	Modeling method
												PSO algorithm	ion ,decision tree
**30**	**Wan2023**	**CT**	**Manual**	**Two**	**3D**	**NR**	**107**	**First order, second order, shape**	**NR**	**No**	**NR**	**ICC, LASSO**	**LR**
**31**	**Wu2022**	**CT**	**Manual**	**Two**	**2D**	**ITK-SNAP**	**1967**	**First order, shape, texture (GLCM, GLDM, GLRLM, GLSZM, NGTDM), high order**	**Pyradiomics**	**Yes**	**Yes**	**ICC, Mann-Whitney U test, LASSO regression, LR**	**LASSO regression**
32	WangT2022	1.5T MRI	Manual	Single	3D	ITK-SNAP	1116	First order, texture (GLCM, GLDM, GLRLM, GLSZM, NGTDM), statistical features derived from LoG filtered domain, wavelet	PyRadiomics	No	No	ROC analysis, nearest neighbor error algorithm	SVM, multivariate LR
**33**	**Lu2022**	**1.5T and 3.0T MRI**	**Manual**	**Two**	**3D**	**ITK-SNAP**	**258**	**First order, shape, GLCM, GLSZM, GLRLM, GLDM**	**Pyradiomics**	**No**	**No**	**ICC, LASSO**	**LASSO**

No.	StudyID	Modality	Segmentation	No of Reviewer	ROI	Segmentation software	No of features extracted	Types of Radiomics feature extracted	Feature extraction software	Features from sites other than ovaries	Features from multiple sites	Feature selection method	Modeling method
**34**	**LiC2022**	**3.0T MRI**	**Manual**	**Two**	**3D**	**ITK-SNAP**	**1316**	**Shape, first order, GLCM, GLRLM, GLSZM, NGTDM, GLDM**	**Pyradiomics**	**No**	**NR**	**ICC, univariate logistic analysis, LASSO**	**LR**
**35**	**Hu2022**	**CT**	**Manual**	**Three**	**2D**	**ITK-SNAP**	**851**	**Shape, histogram, GLCM, GLSZM, GLRLM, NGTDM, GLDM, wavelet**	**Pyradiomics**	**Yes**	**Yes**	**Spearman correlation coefficient, FAMUS, LASSO**	**Cox proportional hazards model**
36	Hong2022	CT	Semi-automatic	Two	3D	3D-slicer	1316	First order, GLCM, GLRLM, GLSZM, NGTDM, GLDM, wavelet, LoG	Pyradiomics	No	No	LASSO	linear combination, multivariate Cox proportional hazards regression

No.	StudyID	Modality	Segmentation	No of Reviewer	ROI	Segmentation software	No of features extracted	Types of Radiomics feature extracted	Feature extraction software	Features from sites other than ovaries	Features from multiple sites	Feature selection method	Modeling method
37	Gao2022	CT	Manual	Two	3D	3D-slicer	107	First order, second order	Pyradiomics	No	No	ICC, LASSO	LR
38	Fotopoulou2022	CT	Manual	Multiple	3D	ITK-SNAP	657	Shape, size, first order, second order, wavelet	TextLAB	No	Yes	Cox regression, LASSO	LASSO
39	Feng2022	CT	Manual	Two	2D	ITK-SNAP	806	Texture, shape, GLCM, GLRLM, GLSZM, GLDM	PyRadiomics	No	No	ICC, AUC	Z-score, PCC, RFE, LR
40	Boehm2022	CT	Manual	Three	3D	ITK-SNAP	600	GLSZM, NGTDM, GLRLM, GLDM, GLCM, wavelet	PyRadiomics	Yes	Yes	Cox regression	Cox model
41	Avesani2022	CT	Manual	Group 1: Single Group 2-4:Two	3D	Group 1: DICOM RT Structure Group 2-	217	Statistical, morphological, textural	MODDICOM	No	No	ANOVA, WMW, PCC, Wilcoxon-Mann-Whitney statistical test, Pearson cross-	LR, RF, SVM, XGBoost

No.	StudyID	Modality	Segmentation	No of Reviewer	ROI	Segmentation software	No of features extracted	Types of Radiomics feature extracted	Feature extraction software	Features from sites other than ovaries	Features from multiple sites	Feature selection method	Modeling method
						4:ITK-SNAP						correlation coefficient	
**42**	**YuXY2021**	**3T MRI**	**Manual**	**Two**	**3D**	**3D Slicer**	**1037**	**Gray histogram, morphological, GLCM, GLRLM, GLSZM**	**PyRadiomics**	**No**	**No**	**ICC, mRMR, LASSO. multi-factor stepwise LR**	**LR**
43	Yi2021	CT	Manual	Two	3D	MaZda	340	Wavelet, gradient, histogram, GLCM, GLRLM	MaZda	No	No	LASSO	LASSO& RF, SVM
**44**	**Song.2.2021**	**3T MRI**	**Manual**	**Two**	**3D**	**ITK-SNAP**	**1130**	**First order, shape, texture, transform**	**PyRadiomics**	**No**	**No**	**ICC, Wilcoxon rank-sum test, LASSO, multivariate LR**	**LR**
**45**	**Liu2021**	**CT**	**Manual**	**Two**	**2D& 3D**	**ITK-SNAP**	**2043**	**Histogram, grayscale interconnected area matrix, grayscale co-occurrence**	**Analysis Kit**	**No**	**No**	**mRMR, LASSO**	**LR**

No.	StudyID	Modality	Segmentation	No of Reviewer	ROI	Segmentation software	No of features extracted	Types of Radiomics feature extracted	Feature extraction software	Features from sites other than ovaries	Features from multiple sites	Feature selection method	Modeling method
								**matrix, morphology, RLM, wavelet, Laplacian**					
**46**	**LiM2021**	**CT**	**Manual**	**Two**	**3D**	**ITK-SNAP**	**653**	**Histograms, GURM, NGDM, GLCM, morphology, RLM**	**AnalysisKit**	**No**	**No**	**Mann-Whitney U test, single-factor LR analysis, mRMR , Multiple LR analysis**	**LR**
47	LiH.1.2021	1.5T/3T MRI	Manual	Single	3D	ITK-SNAP	1046	Original image features, LoG features, wavelet	PyRadiomics	No	No	LASSO	SVM, information-fusion method
48	LiH.2.2021	1.5T/3T MRI	Manual	Single	3D	MITK	1682	Shape, first order, GLCM, GLSZM, GLRLM, NGTDM, GLDM, wavelet	PyRadiomics	No	Yes	ICC, correlation analysis, mRMR, LASSO	LASSO, LR

No.	StudyID	Modality	Segmentation	No of Reviewer	ROI	Segmentation software	No of features extracted	Types of Radiomics feature extracted	Feature extraction software	Features from sites other than ovaries	Features from multiple sites	Feature selection method	Modeling method
49	Chen.1.2021	CT	Manual	Two	3D	ITK-SNAP	696	First order, shape , textural phenotype features, histogram, second-order textural features(GLCM, GLRIM, GLSZM, NGTDM, GLDM), wavelet, local binary patterns	Intelligence Foundry	No	NR	Spearman correlation coefficient, SVM-RFE	LR
50	Chen.2.2021	CT	Manual	Two	3D	ITK-SNAP	696	NR	Intelligence Foundry	No	NR	Spearman correlation coefficient, RF	LR
51	Ai2021	CT	Manual	Single	3D	LIFEx	148	First order(histogram, shape, conventional statistics), second order(GLCM,	LIFEx	No	No	Mann-Whitney U tests, LASSO, Ridge Regression	LASSO regression, LR

No.	StudyID	Modality	Segmentation	No of Reviewer	ROI	Segmentation software	No of features extracted	Types of Radiomics feature extracted	Feature extraction software	Features from sites other than ovaries	Features from multiple sites	Feature selection method	Modeling method
**52**	**Veeraraghavan 2020**	**CT**	**Manual**	**Two**	**3D**	**3D Slicer**	**cluDiss: NR; ARHM: 75[a]**	**NGLDM, GLRLM, GLZLM) cluDiss: Haralick textures, energy, entropy, homogeneity, contrast; ARHM: first order histogram, GLCM, GLSZM, NGTDM, NGLDM, mean values of Sobel, Gabor edges**	**ITK, CERR**	**Yes**	**Yes**	**cluDiss:NR AHRF: Nested cross-validation, SMOTE method, SVM-RFE**	**Elastic net feature selection constraints, SVM**
53	Wei2019	CT	Manual	Two	3D	ITK-SNAP	620	Histogram, shape, textural, wavelet	MATLAB	No	No	LASSO	Cox model
54	Meier2019	CT	Manual	NR	3D	ITK-SNAP	NR	Texture, haralick(GLCM, SE, SCV, SCP)	MATLAB	Yes	Yes	NR	NR

No.	StudyID	Modality	Segmentation	No of Reviewer	ROI	Segmentation software	No of features extracted	Types of Radiomics feature extracted	Feature extraction software	Features from sites other than ovaries	Features from multiple sites	Feature selection method	Modeling method
55	Lu2019	CT	Manual	Two	3D	ITK-SNAP	657	Shape&size, first order , second order, wavelet	TextLAB 2.0	No	No	Cox regression, LASSO	LASSO
56	Rizzo2018	CT	Manual	Single	3D	DICOM RT Structure	1419	Shape, intensity histogram, intensity direct, GLCM, GLRLM, Neighbour Intensity Difference	IBEX	No	No	Test-retest experiments on a phantom, One-way ANOVA, hierarchical clustering procedure, regression models with VIF, multivariate analysis, univariate analysis, stepwise	LR

No.	StudyID	Modality	Segmentation	No of Reviewer	ROI	Segmentation software	No of features extracted	Types of Radiomics feature extracted	Feature extraction software	Features from sites other than ovaries	Features from multiple sites	Feature selection method	Modeling method
												selection procedure	
57	Vargas2017	CT	Manual	NR	3D	3D Slicer	12	First order(mean, SD, kurtosis, skewness), GLCM haralick texture(energy, correlation, contrast, homogeneity), Similarity Level-Area matrix metrics(SE, SCV, SCS, SCP)	NR	Yes	Yes	Decision tree, LASSO	LR

Note: [a] indicates Two kinds of models were discussed and the radiomics features were extracted to establish the two models separately.

Abbreviation:CAD: computer- aided detection, GLDM: gray-level cooccurrence matrix, GLRLM: gray-level run-length matrix, GLSZM: gray-level size zone matrix, NGTDM: neighboring gray tone difference matrix, GLDM: gray-level dependence matrix, RLM: run-length matrix, FFT: Fast Fourier Transform, DCT: Discrete Cosine Transform, NGDM: neighborhood grayscale difference matrix, GURM: gray unicom regional matrix, GLZLM: gray-level zone length matrix , NGLDM: neighborhood gray level dependence matrix, SE: inter-site entropy, SCV: inter-site cluster variance, SCP: inter-site cluster prominence, SCS: inter-site cluster shade, NR:not reported, LASSO:least absolute shrinkage and selection operator, ICC: intraclass correlation coefficient, RF: random forest, PCC: Pearson correlation coefficients, mRMR:minimum Redundancy Maximum Relevance, LR:logistic regression, RFECV: recursive feature elimination algorithm through crossvalidation, ROC:

receiver operating characteristic, ANOVA: analysis of variance, PSO: particle swarm optimization, SVM:support vector machines, KNN: k-nearest neighbor, SVM-RFE: support vector machines-recursive feature elimination, MLP: multi-layer perceptron, XGBoost: eXtreme Gradient Boosting, NB: naïve Bayes, FAMUS: Frequency Appearance in Multiple Univariate preScreening, VIF: variance inflation factors, AHRF: average heterogeneity radiomic features,

Supplementary Table S6. Individual RQS Ratings (M.H./J.R.) and average rating per item

No	StudyID	Image Protocol	Multiple Segmentations	Phantom Study	Multiple Timepoints	Feature Reduction	Non Radiomics	Biological Correlates	Cut Off	Discrimination/ [illegible]	Calibration/Resampling	Prospective	Validation	Gold Standard	Clinical Utility	Cost	Open Science	Total	(%)
1	Zheng2022	1/1	1/1	0/0	0/0	3/3	1/1	0/0	0/0	1/1	0/0	0/0	2/2	2/2	0/0	0/0	0/0	11/11	30.6/30.6
2	Zhang2022	1/1	1/1	0/0	0/0	3/3	1/1	0/0	0/0	1/1	1/1	0/0	2/2	2/2	2/2	0/0	0/0	14/14	38.9/38.9
3	Xu2022	1/1	0/0	0/0	0/0	3/3	1/1	0/0	0/0	2/2	1/1	0/0	2/2	2/2	2/2	0/0	0/0	14/14	38.9/38.9
4	Wei2022	1/1	1/1	0/0	0/0	3/3	1/1	0/0	0/0	2/2	0/0	0/0	4/4	2/2	0/0	0/0	0/0	14/14	38.9/38.9
5	M.Wang2022	1/1	1/1	0/0	0/0	3/3	0/0	0/0	0/0	2/2	0/0	0/0	5/2	2/2	0/0	0/0	0/0	14/11	38.9/30.6
6	Nagawa2022	1/1	1/1	0/0	0/0	3/3	1/1	0/0	0/0	2/2	0/0	0/0	-5/-5	2/2	0/0	0/0	0/0	5/5	13.9/13.9
7	LiuX2022	0/0	0/0	0/0	0/0	3/3	0/0	0/0	0/0	1/1	0/0	0/0	2/2	2/2	0/0	0/0	0/0	8/8	22.2/22.2
8	LiuP2022	1/1	0/0	0/0	0/0	3/3	1/1	0/0	0/0	2/1	0/0	0/0	2/2	2/2	0/0	0/0	0/0	11/10	30.6/27.8

No	StudyID	Image Protocol	Multiple Segmentations	Phantom Study	Multiple Timepoints	Feature Reduction	Non Radiomics	Biological Correlates	Cut Off	Discrimination/ Resampling	Calibration/Resampling	Prospective	Validation	Gold Standard	Clinical Utility	Cost	Open Science	Total	(%)
9	LiS2022	1/1	1/1	0/0	0/0	3/3	1/1	0/0	0/0	1/1	1/1	0/0	2/2	2/2	2/2	0/0	0/0	14/14	38.9/38.9
10	LiJ.1.2022	1/1	1/1	0/0	0/0	3/3	1/1	0/0	0/0	2/2	0/0	0/0	2/2	2/2	0/0	0/0	0/0	12/12	33.3/33.3
11	LiJ.2.2022	1/1	1/1	0/0	0/0	3/3	1/1	0/0	0/0	1/1	1/1	0/0	2/2	2/2	2/2	0/0	0/0	14/14	38.9/38.9
12	Zhu2021	1/1	0/0	0/0	0/0	3/3	1/1	0/0	0/0	1/1	2/2	0/0	2/2	2/2	2/2	0/0	0/0	14/14	38.9/38.9
13	YuXP2021	1/0	1/1	0/0	0/0	3/3	0/0	0/0	0/0	1/1	0/0	0/0	2/2	2/2	0/0	0/0	0/0	10/9	27.8/25.0
14	Ye2021	1/1	0/0	0/0	0/0	3/3	1/1	0/0	0/0	1/1	0/0	0/0	2/2	2/2	0/0	0/0	0/0	10/10	27.8/27.8
15	Song.1.2021	1/1	0/0	0/0	0/0	3/3	0/0	0/0	0/0	2/2	1/1	7/7	2/2	2/2	2/2	0/0	0/0	20/20	55.6/55.6
16	Park2021	1/1	0/0	0/0	0/0	3/3	1/1	0/0	0/0	2/2	0/0	0/0	2/2	2/2	0/0	0/0	1/1	12/12	33.3/33.3
17	LiS2021	1/1	1/1	0/0	0/0	3/3	1/1	0/0	0/0	1/1	0/0	0/0	4/4	2/2	2/2	0/0	0/0	15/15	41.7/41.7

No	StudyID	Image Protocol	Multiple Segmentations	Phantom Study	Multiple Timepoints	Feature Reduction	Non Radiomics	Biological Correlates	Cut Off	Discrimination/ [illegible]	Calibration/Resampling	Prospective	Validation	Gold Standard	Clinical Utility	Cost	Open Science	Total	(%)
18	LiN2021	1/1	1/1	0/0	0/0	3/3	1/1	0/0	0/0	1/1	0/0	0/0	-5/-5	2/2	0/0	0/0	0/0	4/4	11.1/11.1
19	Jian2021	0/0	0/0	0/0	0/0	3/3	0/0	0/0	0/0	1/1	0/0	0/0	5/5	2/2	0/0	0/0	0/0	11/11	30.6/30.6
20	Hu2021	1/1	0/0	0/0	0/0	3/3	1/1	0/0	0/0	1/1	1/1	0/0	2/2	2/2	0/0	0/0	0/0	11/11	30.6/30.6
21	An2021	1/1	1/1	0/0	0/0	3/3	1/1	0/0	0/0	1/2	0/0	0/0	2/2	2/2	0/0	0/0	0/0	11/12	30.6/33.3
22	Qian2020	1/1	1/1	0/0	0/0	3/3	1/1	0/0	0/0	2/2	2/2	0/0	2/2	2/2	2/2	0/0	0/0	16/16	44.4/44.4
23	Lupean2020	1/1	0/0	0/0	0/0	3/3	0/0	0/0	0/0	1/1	0/0	0/0	-5/-5	2/2	0/0	0/0	0/0	2/2	5.6/5.6
24	Li2020	0/0	1/1	0/0	0/0	3/3	1/1	0/0	0/0	1/1	0/0	0/0	5/5	2/2	0/0	0/0	0/0	13/13	36.1/36.1
25	Zhang2019	1/1	0/0	0/0	0/0	3/3	0/0	1/0	1/1	1/2	0/0	0/0	2/2	2/2	0/0	0/0	0/0	11/11	30.6/30.6
26	Rundo2022	1/1	1/1	0/0	0/0	3/3	1/1	0/0	0/0	2/2	0/0	0/0	2/2	2/2	0/0	0/0	0/0	12/12	33.3/33.3

No	StudyID	Image Protocol	Multiple Segmentations	Phantom Study	Multiple Timepoints	Feature Reduction	Non Radiomics	Biological Correlates	Cut Off	Discrimination/ Resampling	Calibration/Resampling	Prospective	Validation	Gold Standard	Clinical Utility	Cost	Open Science	Total	(%)
27	Zargari2018	2/2	0/0	0/0	0/0	3/3	0/0	0/0	0/0	2/2	0/0	0/0	2/2	2/2	0/0	0/0	0/0	11/11	30.6/30.6
28	Danala2017	2/2	0/0	0/0	0/0	3/3	0/0	0/0	0/0	1/1	0/0	0/0	-5/-5	2/2	0/0	0/0	0/0	3/3	8.3/8.3
29	Qiu2016	1/1	0/0	0/0	0/0	-3/-3	0/0	0/0	0/0	1/1	0/0	0/0	-5/-5	2/2	0/0	0/0	0/0	-4/-4	0.0/0.0
30	Wan2023	1/1	1/1	0/0	0/0	3/3	1/1	1/1	1/1	2/2	1/1	0/0	2/2	2/2	2/2	0/0	1/1	18/18	50.0/50.0
31	Wu2022	1/1	1/1	0/0	0/0	3/3	1/1	0/0	0/0	1/1	1/1	0/0	2/2	0/0	2/2	0/0	0/0	12/12	33.3/33.3
32	WangT2022	1/1	0/0	0/0	0/0	3/3	1/1	0/0	0/0	2/2	1/1	0/0	2/2	0/0	2/2	0/0	0/0	12/12	33.3/33.3
33	Lu2022	1/1	1/1	0/0	0/0	3/3	1/1	0/0	0/0	2/2	2/2	0/0	2/2	0/0	2/2	0/0	0/0	14/14	38.9/38.9
34	LiC2022	1/1	1/1	0/0	0/0	3/3	1/1	0/0	0/0	1/1	1/1	0/0	2/2	0/0	2/2	0/0	0/0	12/12	33.3/33.3
35	Hu2022	0/0	1/1	0/0	0/0	3/3	0/1	0/0	0/0	2/1	1/1	0/0	4/4	0/0	0/0	0/0	0/0	11/11	30.6/30.6

No	StudyID	Image Protocol	Multiple Segmentations	Phantom Study	Multiple Timepoints	Feature Reduction	Non Radiomics	Biological Correlates	Cut Off	Discrimination/ [illegible]	Calibration/Resampling	Prospective	Validation	Gold Standard	Clinical Utility	Cost	Open Science	Total	(%)
36	Hong2022	1/1	1/1	0/0	0/0	3/3	1/1	0/0	0/0	1/1	0/0	0/0	3/3	0/0	0/0	0/0	0/0	10/10	27.8/27.8
37	Gao2022	1/1	1/1	0/0	0/0	3/3	0/0	1/1	0/0	2/2	1/1	0/0	2/2	0/0	2/2	0/0	1/1	14/14	38.9/38.9
38	Fotopoulou2022	1/0	1/1	0/0	0/0	3/3	0/1	1/1	1/1	1/1	0/0	0/0	4/4	0/0	0/0	0/0	1/1	13/13	36.1/36.1
39	Feng2022	1/1	1/1	0/0	0/0	3/3	0/0	1/1	0/0	1/1	0/0	0/0	2/2	0/0	0/0	0/0	1/1	10/10	27.8/27.8
40	Boehm2022	2/2	1/1	0/0	0/0	3/3	1/1	0/0	0/0	1/2	0/0	0/0	2/2	0/0	0/0	0/0	2/2	12/13	33.3/36.1
41	Avesani2022	0/0	1/1	0/0	0/0	3/3	1/1	1/1	0/0	2/2	0/0	0/0	4/4	0/2	0/0	0/0	0/0	12/14	33.3/38.9
42	YuXY2021	1/1	1/1	0/0	0/0	3/3	1/1	0/0	0/0	1/1	1/1	0/0	-5/-5	2/2	2/2	0/0	0/0	7/7	19.4/19.4
43	Yi2021	1/1	1/1	0/0	0/0	3/3	1/1	1/1	0/0	1/1	1/1	0/0	2/2	0/0	2/2	0/0	0/0	13/13	36.1/36.1
44	Song.2.2021	1/1	1/1	0/0	0/0	3/3	1/1	0/0	0/0	2/2	1/1	7/7	2/2	2/2	2/2	0/0	0/0	22/22	61.1/61.1

No	StudyID	Image Protocol	Multiple Segmentations	Phantom Study	Multiple Timepoints	Feature Reduction	Non Radiomics	Biological Correlates	Cut Off	Discrimination/ [illegible]	Calibration/Resampling	Prospective	Validation	Gold Standard	Clinical Utility	Cost	Open Science	Total	(%)
45	Liu2021	1/1	1/1	0/0	0/0	3/3	0/0	1/1	0/0	1/1	0/0	0/0	2/2	2/2	2/2	0/0	0/0	13/13	36.1/36.1
46	LiM2021	1/1	1/1	0/0	0/0	3/3	1/1	1/1	0/0	2/2	0/0	0/0	2/2	2/2	0/0	0/0	0/0	13/13	36.1/36.1
47	LiH.1.2021	1/1	0/0	0/0	0/0	3/3	1/1	0/0	1/1	2/2	0/0	0/0	2/2	0/0	0/0	0/0	0/0	10/10	27.8/27.8
48	LiH.2.2021	1/1	0/0	0/0	0/0	3/3	1/1	0/0	0/0	1/2	0/0	0/0	2/2	0/0	0/0	0/0	0/0	8/9	22.2/25
49	Chen.1.2021	1/1	1/1	0/0	0/0	3/3	1/1	0/0	0/0	1/1	1/1	0/0	2/2	0/0	2/2	0/0	0/0	12/12	33.3/33.3
50	Chen.2.2021	1/1	1/1	0/0	0/0	3/3	1/1	0/0	0/0	2/2	1/1	0/0	2/2	2/2	2/2	0/0	0/0	15/15	41.7/41.7
51	Ai2021	1/1	0/0	0/0	0/0	3/3	1/1	0/0	0/0	1/1	0/0	0/0	2/2	0/0	0/0	0/0	0/0	8/8	22.2/22.2
52	Veeraraghavan2020	1/1	1/1	1/1	0/0	3/3	1/1	1/1	0/1	1/2	0/0	0/0	3/3	0/0	0/0	0/0	0/0	12/14	33.3/38.9
53	Wei2019	1/1	1/1	0/0	0/0	3/3	1/1	0/0	1/1	2/2	1/1	0/0	4/4	0/0	0/0	0/0	0/0	14/14	38.9/38.9

No	StudyID	Image Protocol	Multiple Segmentations	Phantom Study	Multiple Timepoints	Feature Reduction	Non Radiomics	Biological Correlates	Cut Off	Discrimination/ Resampling	Calibration/Resampling	Prospective	Validation	Gold Standard	Clinical Utility	Cost	Open Science	Total	(%)
54	Meier2019	1/1	0/0	0/0	0/0	-3/-3	0/0	1/1	0/1	0/1	0/0	0/0	-5/-5	2/0	0/0	0/0	0/0	-4/-4	0.0/0.0
55	Lu2019	0/0	1/1	1/1	0/0	3/3	1/1	1/1	0/0	1/1	0/0	0/0	5/5	0/0	0/0	0/0	2/1	15/14	41.7/38.9
56	Rizzo2018	0/0	0/0	1/1	0/0	3/3	1/1	1/1	1/1	1/1	0/0	0/0	-5/-5	0/0	0/0	0/0	0/0	3/3	8.3/8.3
57	Vargas2017	0/0	0/0	0/0	0/0	3/3	0/0	1/0	1/0	2/2	0/0	0/0	-5/2	0/0	0/0	0/0	0/0	2/7	5.6/19.4
	Average	0.89	0.63	0.05	0	2.79	0.72	0.23	0.13	1.42	0.42	0.25	1.39	1.26	0.70	0	0.15	11.04	30.7
	Defined range	0 – 2	0 – 1	0 – 1	0 – 1	-3 – 3	0 – 1	0 – 1	0 – 1	0 – 2	0 – 2	0 – 7	-5 – 5	0 – 2	0 – 2	0 – 1	0 – 4	-8-36	0-100

Total points: -8 to 0 = 0%, 36=100%

Supplementary Table S7. QUADAS-2 assessment for each study

StudyID	Risk of Bias				Applicability Concern		
	Patient Selection	Index Test	Reference Standard	Flow and Timing	Patient Selection	Index Test	Reference Standard
Zheng2022	☹ ☺ ☺	? ?	☺ ☺	☺ ☺	☺ ☺	☺ ☺	☺ ☺
Zhang2022	☺ ☺	☺ ☺	☺ ☺	☺ ☺	☺ ☺	☺ ☺	☺ ☺
Xu2022	☹ ☺ ☺	☺ ☺	☺ ☺	☺ ☺	? ☺ ☺	☺ ☺	☺ ☺
Wei2022	☺ ☺	☺ ☺	☺ ☺	☺ ☺	☺ ☺	☺ ☺	☺ ☺
M.Wang2022	☺ ☺	☺ ☺	☺ ☺	☺ ☺	☺ ☺	☺ ☺	☺ ☺
Nagawa2022	☺ ☺	☺ ☺	☺ ☺	☺ ☺	? ?	☺ ☺	☺ ☺
LiuX2022	☺ ☺	☺ ☺	☺ ☺	? ?	☺ ☺	☺ ☺	☺ ☺
LiuP2022	☹ ☹	☺ ☺	☺ ☺	? ?	☹ ☹	☺ ☺	☺ ☺
LiS2022	☺ ☺	? ☺ ☺	☺ ☺	☺ ☺	☺ ☺	☺ ☺	☺ ☺
LiJ.1.2022	☺ ☺	☺ ☺	☺ ☺	☺ ☺	☺ ☺	☺ ☺	☺ ☺
LiJ.2.2022	☺ ☺	☺ ☺	☺ ? ?	☺ ☺	☺ ☺	☺ ☺	☺ ☺
Zhu2021	☺ ? ☺	☺ ☺	☺ ☺	☺ ☺	☺ ☺	☺ ☺	☺ ☺
YuXP2021	☺ ☺	☹ ☺ ☺	☺ ☺	? ?	☺ ☺	☺ ☺	☺ ☺
Ye2021	☺ ? ☺	☺ ☺	☺ ☺	? ?	☺ ☺	☺ ☺	☺ ☺

StudyID	Risk of Bias				Applicability Concern		
	Patient Selection	Index Test	Reference Standard	Flow and Timing	Patient Selection	Index Test	Reference Standard
Song.1.2021	☺ ☺	☺ ☺	☺ ☺	☺ ☺	☺ ☺	☺ ☺	☺ ☺
Park2021	☺ ☺	☺ ☺	☺ ☺	☹ ☹	☺ ☺	☺ ☺	☺ ☺
LiS2021	☺ ? ☺	☺ ☺	☺ ☺	☺ ☺	☺ ☺	☺ ☺	☺ ☺
LiN2021	☺ ☺	☺ ☺	☺ ☺	? ?	☺ ☺	☺ ☺	☺ ☺
Jian2021	☺ ☺	☺ ☺	☺ ☺	☺ ☺	☺ ☺	☺ ☺	☺ ☺
Hu2021	☺ ☺	☺ ? ☺	☺ ☺	? ?	☺ ☺	? ☺ ☺	☺ ☺
An2021	☺ ☺	☺ ☺	☺ ☺	☺ ? ?	☹ ☹	☺ ☺	☺ ☺
Qian2020	? ☹ ☹	☺ ☺	☺ ☺	☺ ☺	☹ ☹	☺ ☺	☺ ☺
Lupean2020	☹ ☹	? ?	? ☹ ☹	☹ ☹	☹ ☹	? ?	? ?
Li2020	☺ ☺	☺ ☺	☺ ☺	☺ ☺	☺ ☺	? ☺ ☺	☺ ☺
Zhang2019	☺ ☺	☺ ☺	☺ ☺	? ?	? ☺ ☺	☺ ☺	☺ ☺
Rundo2022	☺ ☺	? ?	? ?	☺ ☺	☺ ☺	☺ ☺	☺ ☺
Zargari2018	☺ ☺	☺ ☺	☺ ☺	☺ ☺	☹ ☹	☺ ☺	☺ ☺
Danala2017	☺ ☺	☺ ☺	? ☺ ☺	☺ ☺	☹ ☹	☺ ☺	☺ ☺

StudyID	Risk of Bias				Applicability Concern		
	**Patient Selection**	**Index Test**	**Reference Standard**	**Flow and Timing**	**Patient Selection**	**Index Test**	**Reference Standard**
Qiu2016	☺ ☺	☺ ☺	? ?	☺ ☺	☹ ☹	☺ ☺	☺ ☺
Wan2023	☺ ☺	☹ ☹	☺ ☺	☹ ? ?	☺ ☺	☺ ☺	☺ ☺
Wu2022	☺ ☺	☺ ☺	☺ ☺	☺ ☺	? ?	☺ ☺	☺ ☺
WangT2022	☺ ? ☺	☺ ☺	☺ ☺	☺ ☺	☺ ☺	☺ ☺	☺ ☺
Lu2022	☺ ☺	☺ ☺	☺ ☺	☺ ☺	☺ ☺	☺ ☺	☺ ☺
LiC2022	☺ ☺	☺ ☺	☺ ☺	☺ ? ?	☺ ☺	☺ ☺	☺ ☺
Hu2022	☺ ☺	☺ ☺	☺ ☺	☺ ☺	☺ ☺	☺ ☺	☺ ☺
Hong2022	☺ ☺	☺ ☺	☺ ☺	☺ ☺	☺ ☺	☺ ☺	☺ ☺
Gao2022	☺ ☺	☺ ☺	☺ ☺	? ?	? ☹ ☹	☺ ☺	☺ ☺
Fotopoulou2022	☺ ☺	☹ ☹	☺ ☺	☺ ☺	☺ ☺	☺ ☺	☺ ☺
Feng2022	☹ ☹	? ☺ ☺	? ?	? ?	☹ ☹	☺ ☺	? ?
Boehm2022	☹ ☹	☺ ☺	? ?	? ☺ ☺	☺ ☺	☺ ☺	☺ ☺
Avesani2022	? ?	☺ ☺	☺ ☺	☺ ☺	☹ ☺ ☹	☺ ☺	☺ ☺
YuXY2021	☹ ☹	☺ ☺	☺ ☺	☺ ☺	☹ ☹	☺ ☺	☺ ☺

StudyID	Risk of Bias				Applicability Concern		
	Patient Selection	Index Test	Reference Standard	Flow and Timing	Patient Selection	Index Test	Reference Standard
Yi2021	☺ ☺	☺ ☺	☺ ☺	☺ ☺	☺ ☺	☺ ☺	☺ ☺
Song.2.2021	☺ ☹ ☺	☺ ☺	☺ ☺	☺ ☺	? ☹ ☹	☺ ☺	☺ ☺
Liu2021	☺ ☹ ☹	☺ ☺	☺ ☺	☺ ☺	☹ ☹	☺ ☺	☺ ☺
LiM2021	☹ ☹	☺ ☺	☺ ☺	☺ ☺	? ?	☺ ☺	☺ ☺
LiH.1.2021	☺ ☺	☺ ☺	☺ ☺	☺ ☺	☺ ☺	☺ ☺	☺ ☺
LiH.2.2021	☺ ☺	☺ ☺	☺ ☺	? ☺ ☺	☺ ☺	☺ ☺	? ?
Chen.1.2021	☺ ☺	☺ ☹ ☹	☺ ☺	☺ ☺	☺ ☺	☺ ☺	☺ ☺
Chen.2.2021	☺ ☺	☺ ☺	☺ ☺	☺ ☺	☺ ☺	☺ ☺	☺ ☺
Ai2021	☺ ? ☺	☺ ☺	☺ ☺	☺ ☺	? ☺ ☺	☺ ☺	☺ ☺
Veeraraghavan2020	☺ ☺	☹ ☹	☺ ☺	☺ ☺	☺ ☺	☺ ☺	☹ ? ?
Wei2019	☹ ☹	☹ ☹	☺ ☺	☺ ☺	? ?	☺ ☺	☺ ? ☺
Meier2019	☹ ☹	☺ ☺	☺ ☺	☺ ☺	☺ ☺	☺ ☺	☺ ☺
Lu2019	☹ ☺ ☺	☺ ☺	☺ ☺	☹ ☹	☺ ☺	☺ ☺	☺ ☺
Rizzo2018	☹ ☹	☺ ☺	☺ ☺	☹ ☺ ☺	☺ ? ☺	☺ ☺	☺ ☺

StudyID	Risk of Bias				Applicability Concern		
	Patient Selection	Index Test	Reference Standard	Flow and Timing	Patient Selection	Index Test	Reference Standard
Vargas2017	☺ ☺<br>80.7%	? ☹ ☹<br>89.5%	☺ ☺<br>94.7%	☺ ☺<br>89.5%	☺ ☺<br>87.7%	☺ ☺<br>96.5%	☺ ☺<br>96.5%

Note: Assessments are presented as MH/JR/YH. ☺, high;☹ , low; ?, unclear. QUADAS, quality assessment diagnostic accuracy studies

Supplementary Figure S1. Forrest plot of pooled sensitivity and specificity of radiomics models in a) differentiation diagnosis, b) prognosis prediction.

a)

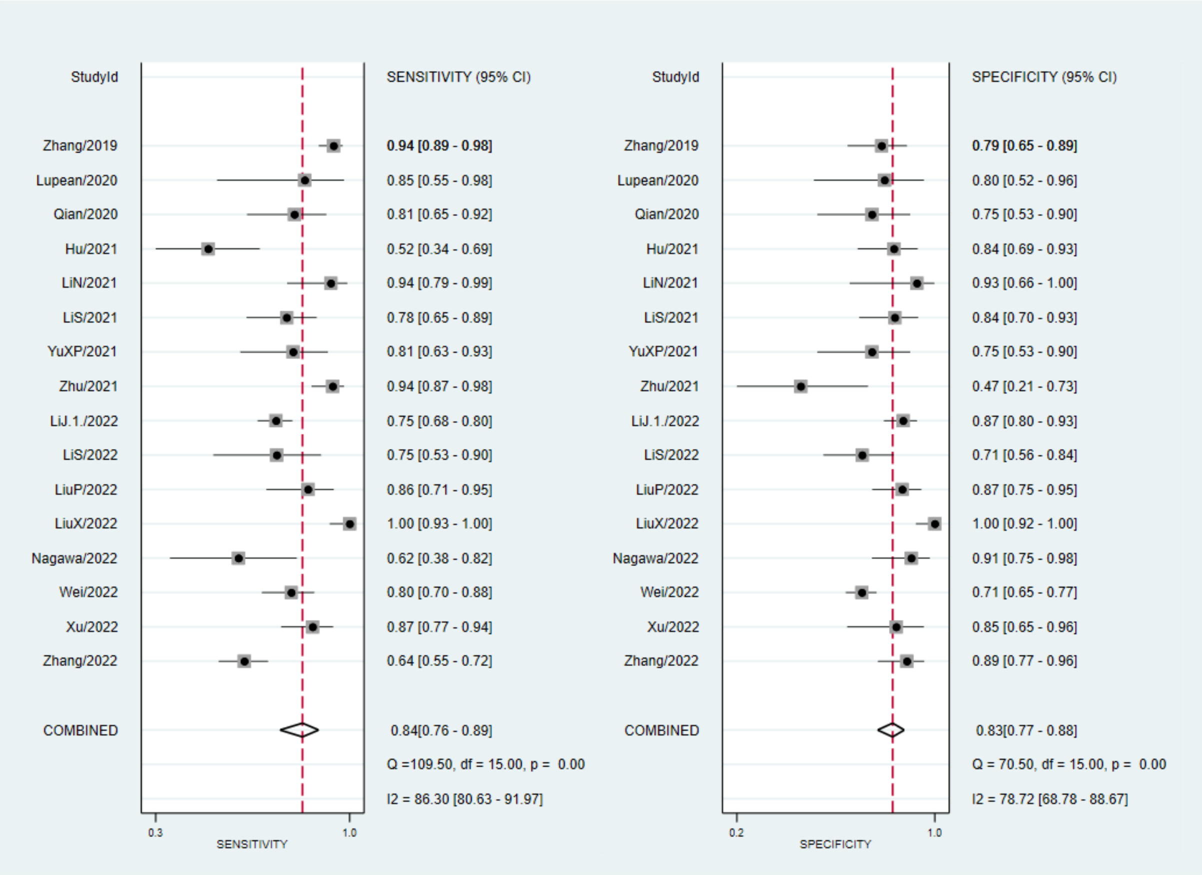

b)

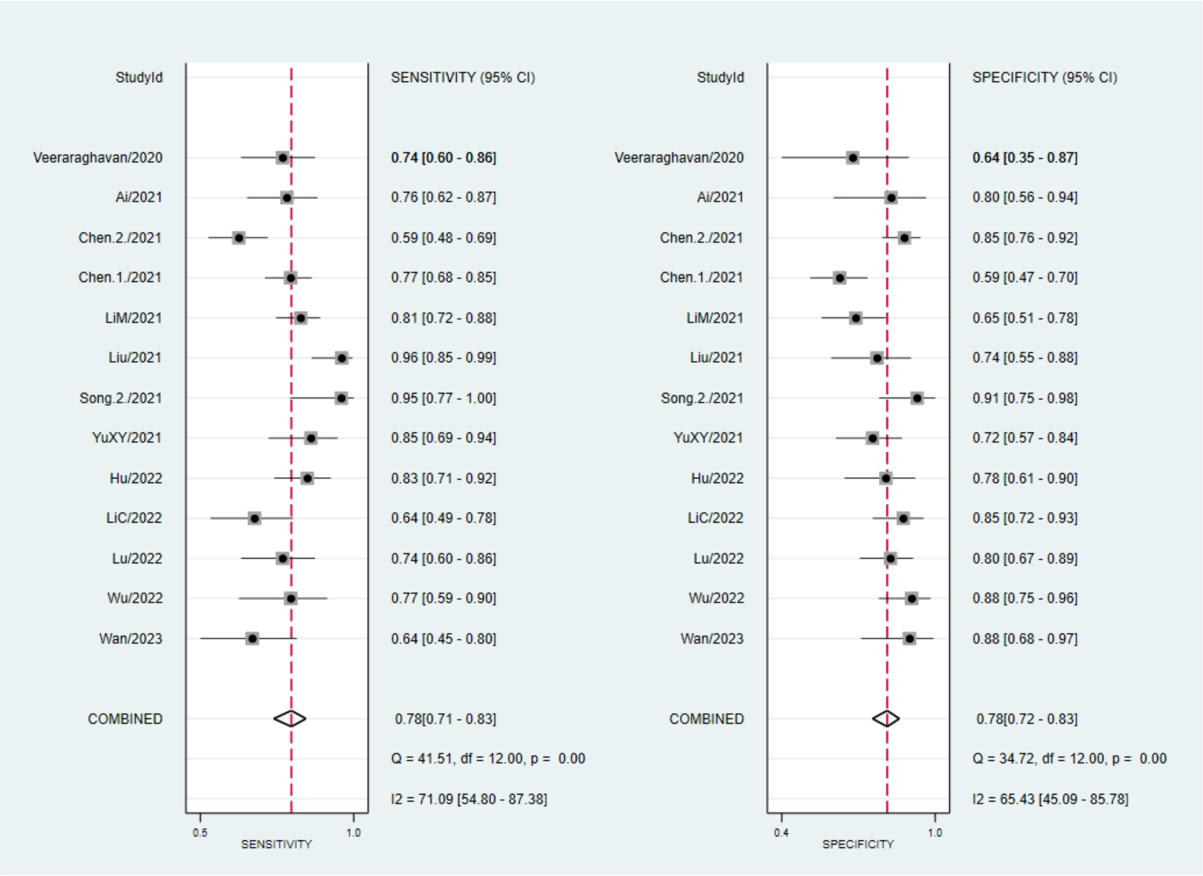
StudyId
SENSITIVITY (95% CI)
Veeraraghavan/2020 0.74 [0.60 - 0.86]
Ai/2021 0.76 [0.62 - 0.87]
Chen.2./2021 0.59 [0.48 - 0.69]
Chen.1./2021 0.77 [0.68 - 0.85]
LiM/2021 0.81 [0.72 - 0.88]
Liu/2021 0.96 [0.85 - 0.99]
Song.2./2021 0.95 [0.77 - 1.00]
YuXY/2021 0.85 [0.69 - 0.94]
Hu/2022 0.83 [0.71 - 0.92]
LiC/2022 0.64 [0.49 - 0.78]
Lu/2022 0.74 [0.60 - 0.86]
Wu/2022 0.77 [0.59 - 0.90]
Wan/2023 0.64 [0.45 - 0.80]
COMBINED 0.78[0.71 - 0.83]
Q = 41.51, df = 12.00, p = 0.00
I2 = 71.09 [54.80 - 87.38]
0.5
1.0
SENSITIVITY
StudyId
SPECIFICITY (95% CI)
Veeraraghavan/2020 0.64 [0.35 - 0.87]
Ai/2021 0.80 [0.56 - 0.94]
Chen.2./2021 0.85 [0.76 - 0.92]
Chen.1./2021 0.59 [0.47 - 0.70]
LiM/2021 0.65 [0.51 - 0.78]
Liu/2021 0.74 [0.55 - 0.88]
Song.2./2021 0.91 [0.75 - 0.98]
YuXY/2021 0.72 [0.57 - 0.84]
Hu/2022 0.78 [0.61 - 0.90]
LiC/2022 0.85 [0.72 - 0.93]
Lu/2022 0.80 [0.67 - 0.89]
Wu/2022 0.88 [0.75 - 0.96]
Wan/2023 0.88 [0.68 - 0.97]
COMBINED 0.78[0.72 - 0.83]
Q = 34.72, df = 12.00, p = 0.00
I2 = 65.43 [45.09 - 85.78]
0.4
1.0
SPECIFICITY

Supplementary Figure S2. Forrest plot of pooled positive likelihood ratio and negative likelihood ratio of radiomics models in a) differentiation diagnosis, b) prognosis prediction.

a)

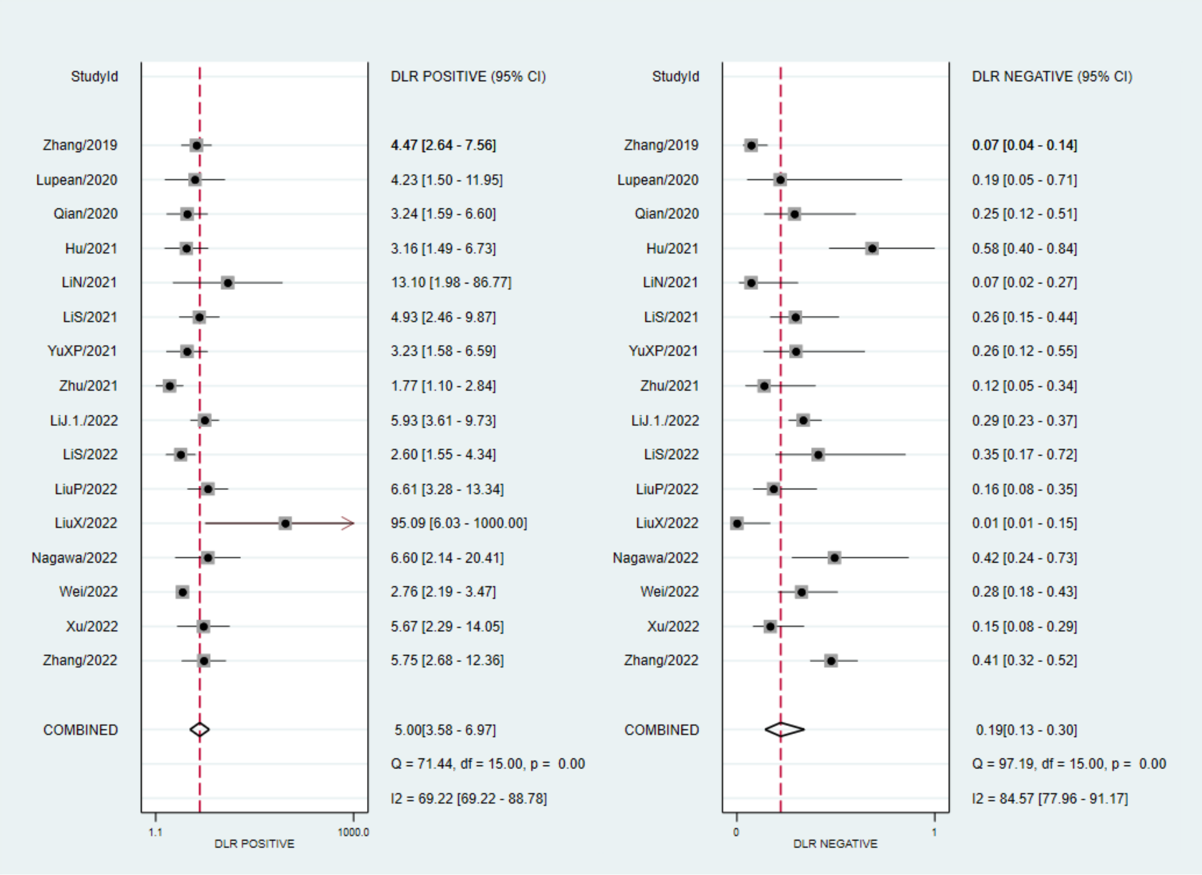

b)

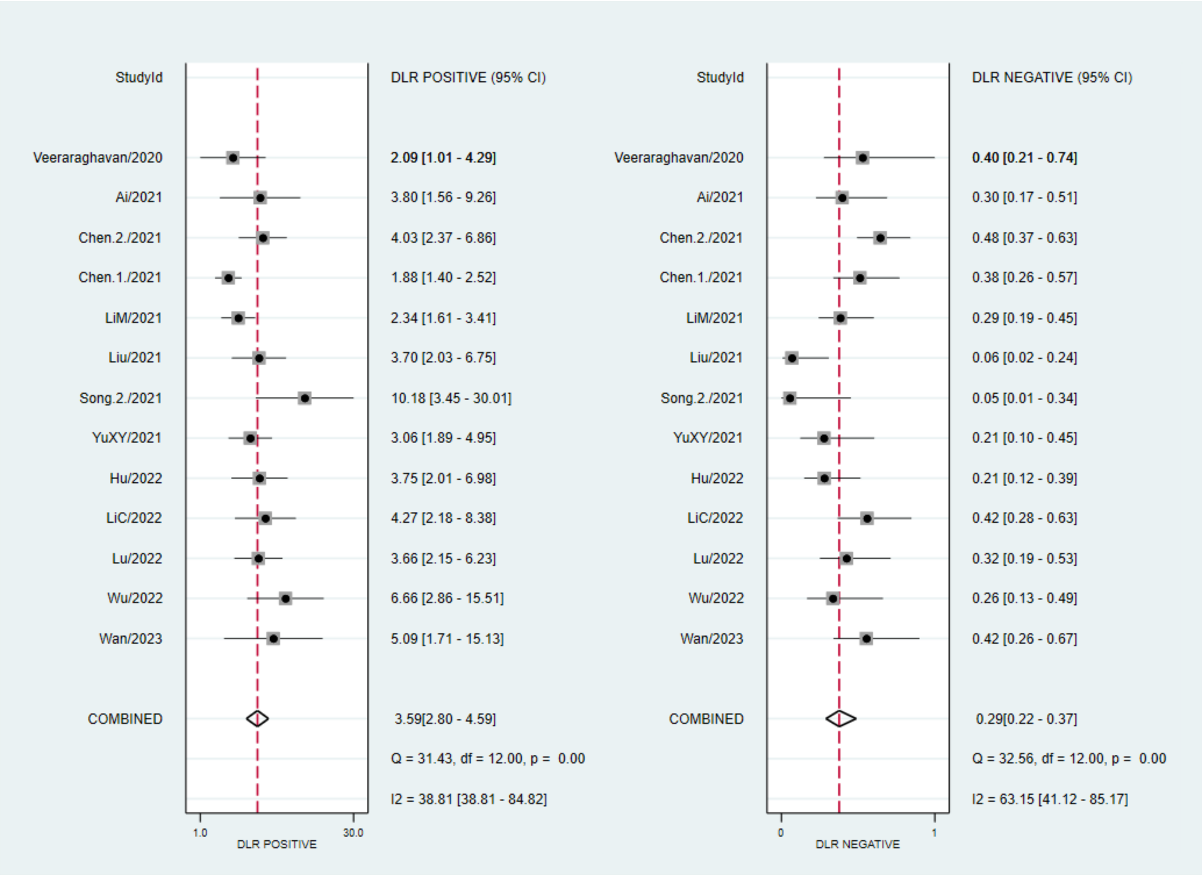
StudyId
DLR POSITIVE (95% CI)
Veeraraghavan/2020
2.09 [1.01 - 4.29]
Ai/2021
3.80 [1.56 - 9.26]
Chen.2./2021
4.03 [2.37 - 6.86]
Chen.1./2021
1.88 [1.40 - 2.52]
LiM/2021
2.34 [1.61 - 3.41]
Liu/2021
3.70 [2.03 - 6.75]
Song.2./2021
10.18 [3.45 - 30.01]
YuXY/2021
3.06 [1.89 - 4.95]
Hu/2022
3.75 [2.01 - 6.98]
LiC/2022
4.27 [2.18 - 8.38]
Lu/2022
3.66 [2.15 - 6.23]
Wu/2022
6.66 [2.86 - 15.51]
Wan/2023
5.09 [1.71 - 15.13]
COMBINED
3.59[2.80 - 4.59]
Q = 31.43, df = 12.00, p = 0.00
I2 = 38.81 [38.81 - 84.82]
1.0
30.0
DLR POSITIVE
StudyId
DLR NEGATIVE (95% CI)
Veeraraghavan/2020
0.40 [0.21 - 0.74]
Ai/2021
0.30 [0.17 - 0.51]
Chen.2./2021
0.48 [0.37 - 0.63]
Chen.1./2021
0.38 [0.26 - 0.57]
LiM/2021
0.29 [0.19 - 0.45]
Liu/2021
0.06 [0.02 - 0.24]
Song.2./2021
0.05 [0.01 - 0.34]
YuXY/2021
0.21 [0.10 - 0.45]
Hu/2022
0.21 [0.12 - 0.39]
LiC/2022
0.42 [0.28 - 0.63]
Lu/2022
0.32 [0.19 - 0.53]
Wu/2022
0.26 [0.13 - 0.49]
Wan/2023
0.42 [0.26 - 0.67]
COMBINED
0.29[0.22 - 0.37]
Q = 32.56, df = 12.00, p = 0.00
I2 = 63.15 [41.12 - 85.17]
0
1
DLR NEGATIVE

Supplementary Figure S3. HSROC curve of the performance for radiomics models in a) differentiation diagnosis,b) prognosis prediction..

a)

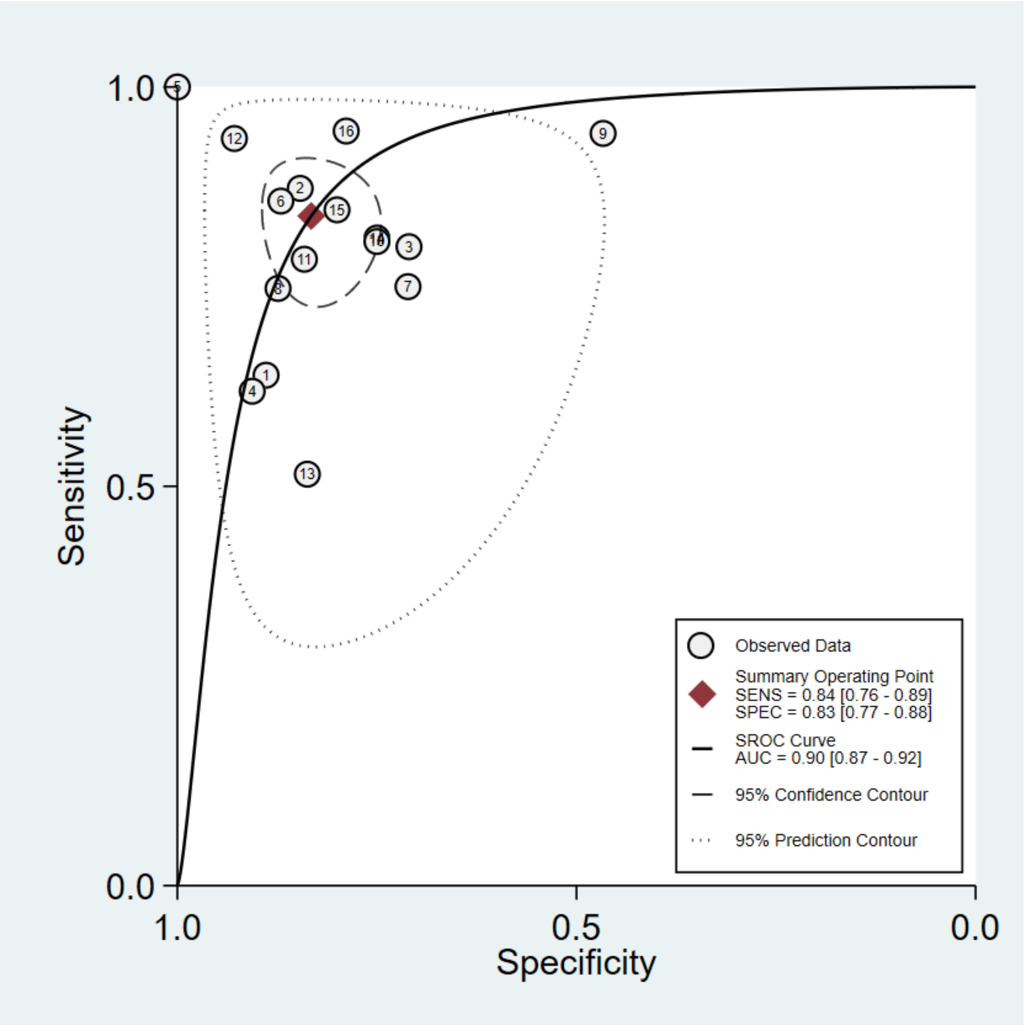

1.0
0.5
0.0
Sensitivity
1.0
0.5
0.0
Specificity
Observed Data
Summary Operating Point
SENS = 0.84 [0.76 - 0.89]
SPEC = 0.83 [0.77 - 0.88]
SROC Curve
AUC = 0.90 [0.87 - 0.92]
95% Confidence Contour
95% Prediction Contour

b)

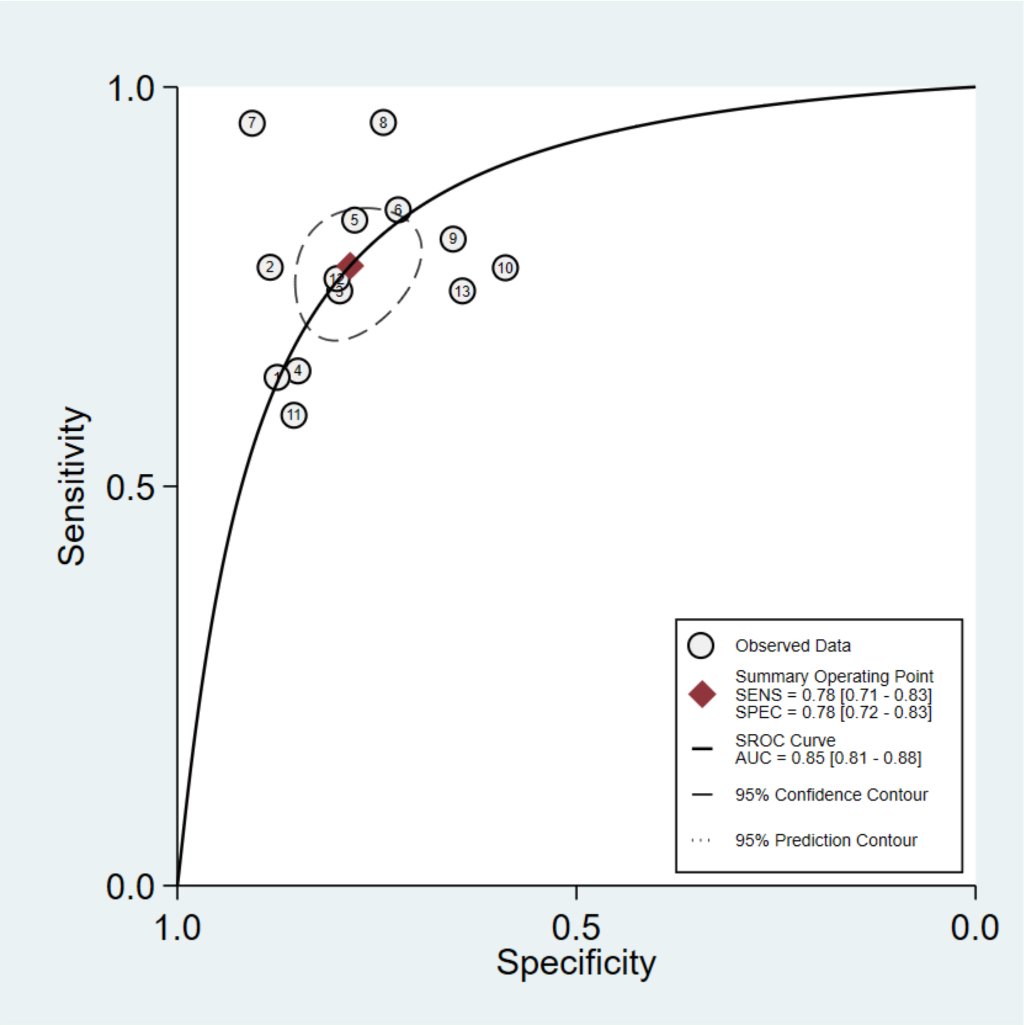

Supplementary Figure S4. Deeks funnel plot of studies included in the meta-analysis about a) differentiation diagnosis, b) prognosis prediction.

a)

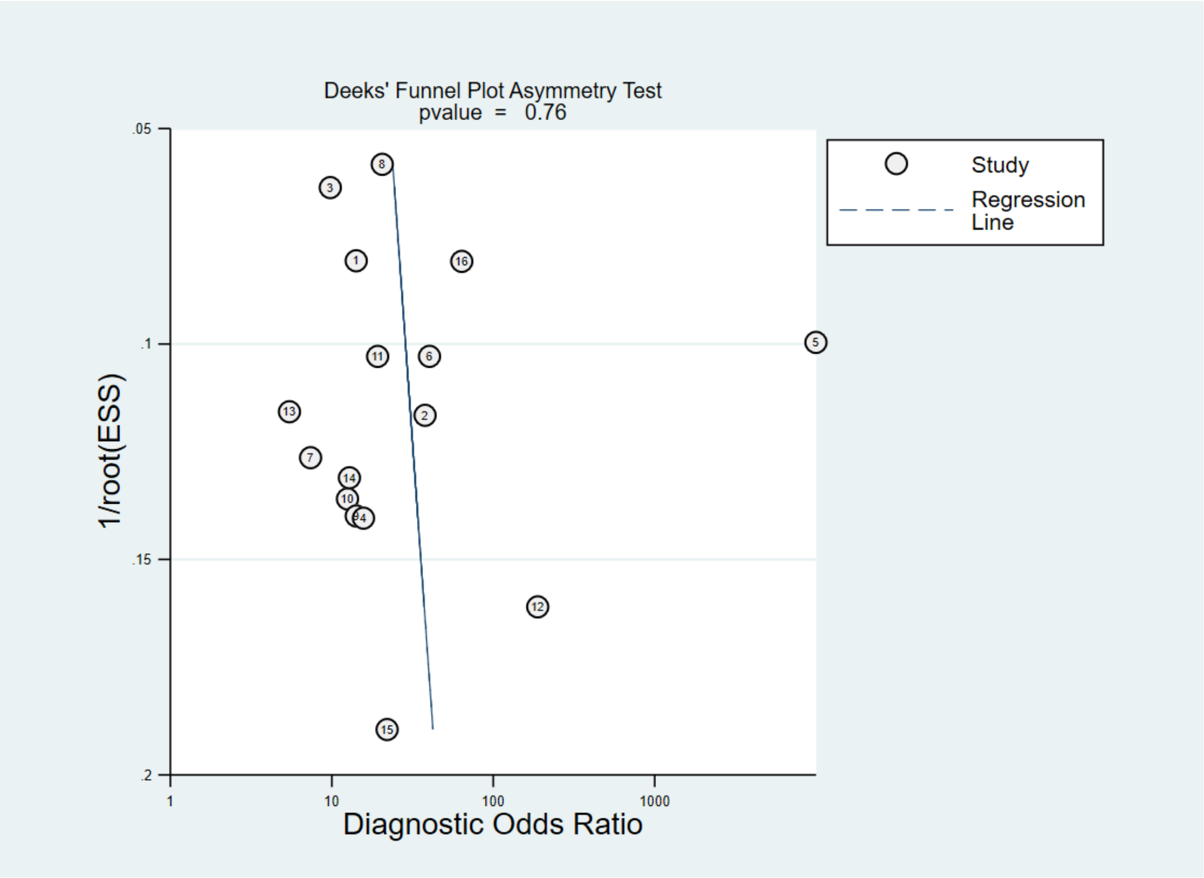

b)

Insights Imaging (2023) Huang ML, Ren J, Jin ZY et al.

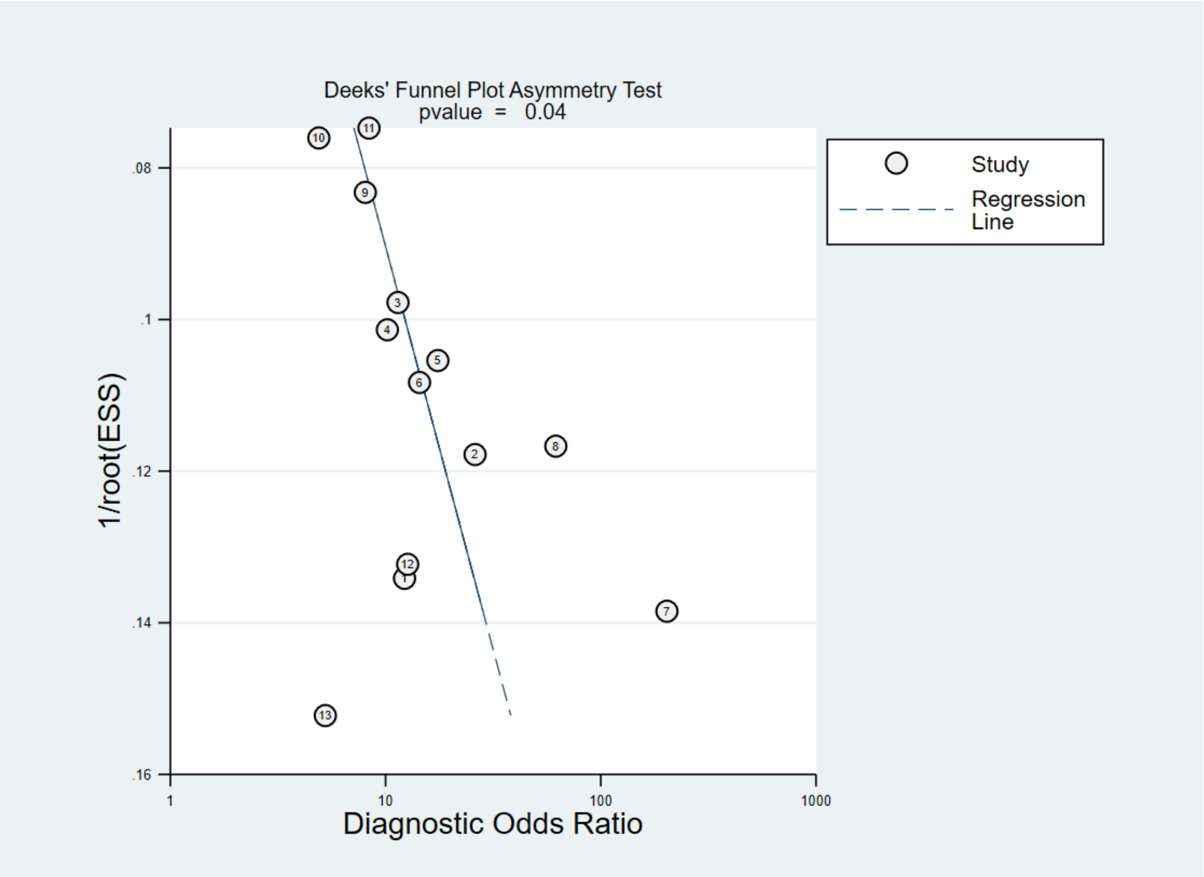

Insights Imaging (2023) Huang ML, Ren J, Jin ZY et al.

Supplementary Figure S5. Univariable meta-regression analysis based on patient, imaging modality, type of ROI and type of features of a) differentiation diagnosis, b) prognosis prediction.

a)

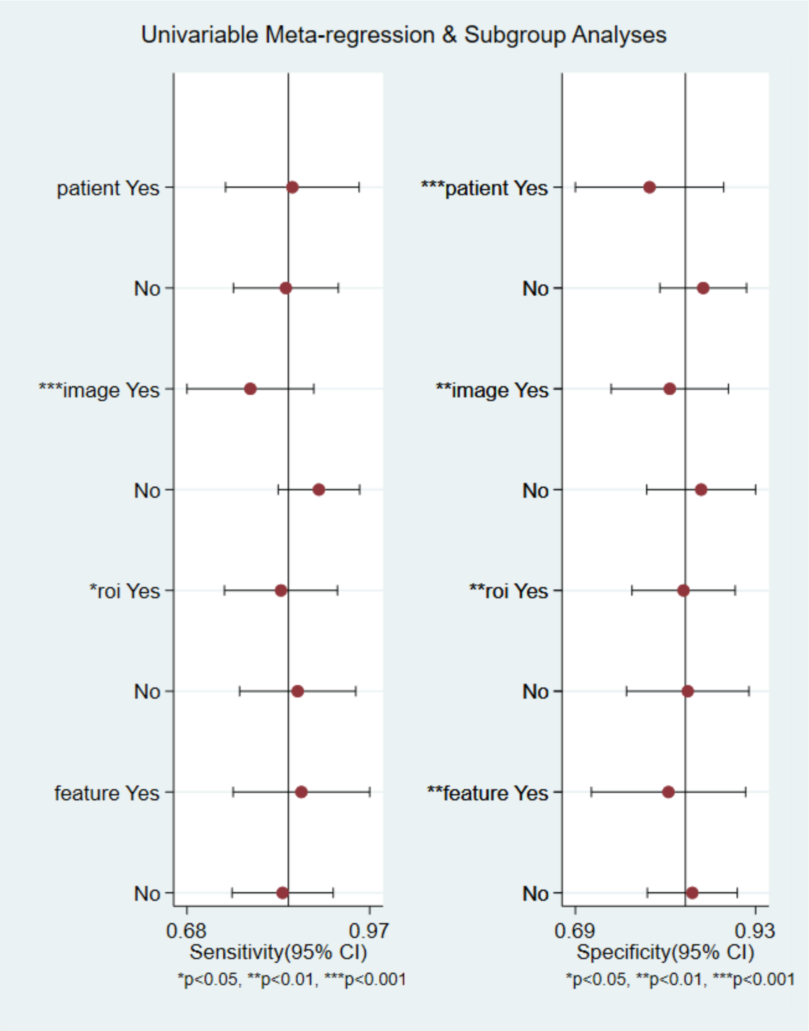

b)

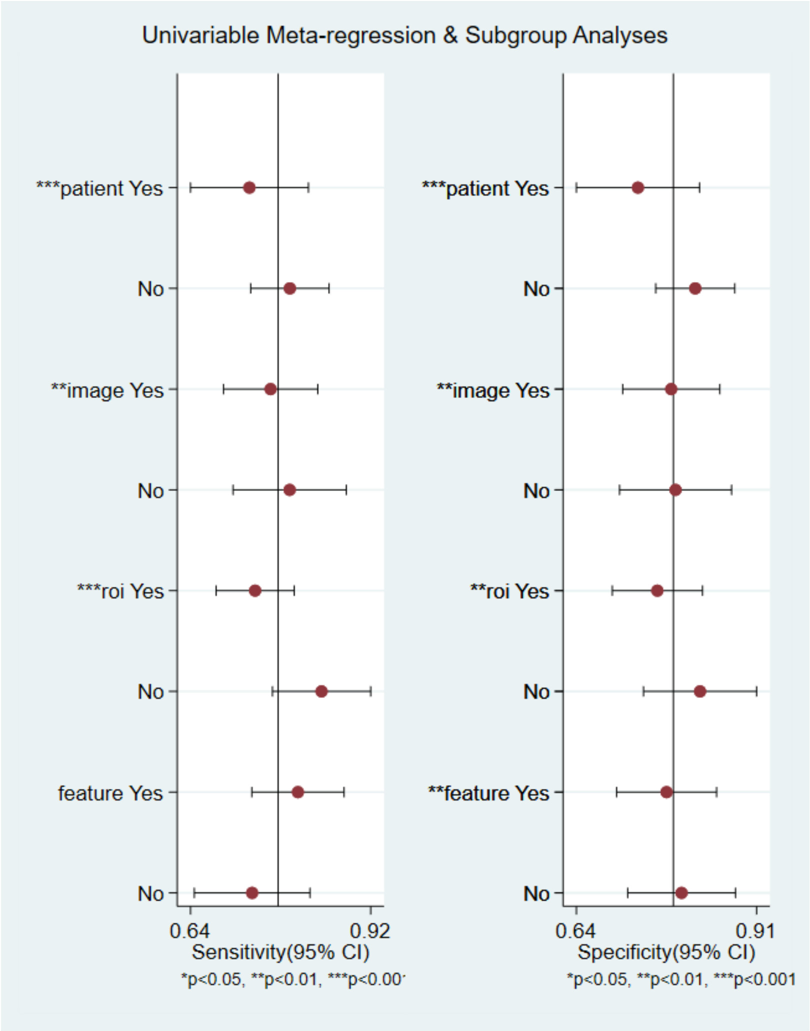

Univariable Meta-regression & Subgroup Analyses
***patient Yes
No
**image Yes
No
***roi Yes
No
feature Yes
No
0.64
0.92
Sensitivity(95% CI)
*p<0.05, **p<0.01, ***p<0.00
***patient Yes
No
**image Yes
No
**roi Yes
No
**feature Yes
No
0.64
0.91
Specificity(95% CI)
*p<0.05, **p<0.01, ***p<0.001

References

1. Zheng Y, Wang H, Li Q, Sun H, Guo L. Discriminating Between Benign and Malignant Solid Ovarian Tumors Based on Clinical and Radiomic Features of MRI. Acad Radiol. 2022.
2. Zhang A, Hu Q, Ma Z, Song J, Chen T. Application of enhanced computed tomography-based radiomics nomogram analysis to differentiate metastatic ovarian tumors from epithelial ovarian tumors. J Xray Sci Technol. 2022; 30(6):1185-99.
3. Xu Y, Luo HJ, Ren J, Guo LM, Niu J, Song X. Diffusion-weighted imaging-based radiomics in epithelial ovarian tumors: Assessment of histologic subtype. Frontiers in Oncology. 2022; 12.
4. Wei M, Zhang Y, Bai G, et al. T2-weighted MRI-based radiomics for discriminating between benign and borderline epithelial ovarian tumors: a multicenter study. Insights Imaging. 2022; 13(1):130.
5. Wang M, Perucho JAU, Hu Y, et al. Computed Tomographic Radiomics in Differentiating Histologic Subtypes of Epithelial Ovarian Carcinoma. JAMA Netw Open. 2022; 5(12):e2245141.
6. Nagawa K, Kishigami T, Yokoyama F, et al. Diagnostic utility of a conventional MRI-based analysis and texture analysis for discriminating between ovarian thecoma-fibroma groups and ovarian granulosa cell tumors. J Ovarian Res. 2022; 15(1):65.
7. Liu X, Wang T, Zhang G, et al. Two-dimensional and three-dimensional T2 weighted imaging-based radiomic signatures for the preoperative discrimination of ovarian borderline tumors and malignant tumors. Journal of ovarian research. 2022; 15(1):22-.
8. Liu P, Liang X, Liao S, Lu Z. Pattern Classification for Ovarian Tumors by Integration of Radiomics and Deep Learning Features. Curr Med Imaging. 2022; 18(14):1486-502.
9. Li S, Liu J, Xiong Y, et al. Application Values of 2D and 3D Radiomics Models Based on CT Plain Scan in Differentiating Benign from Malignant Ovarian Tumors. Biomed Res Int. 2022; 2022:5952296.
10. Li J, Zhang T, Ma J, Zhang N, Zhang Z, Ye Z. Machine-learning-based contrast-enhanced computed tomography radiomic analysis for categorization of ovarian tumors. Front Oncol. 2022; 12:934735.
11. Li J, Li X, Ma J, Wang F, Cui S, Ye Z. Computed tomography-based radiomics machine learning classifiers to differentiate type I and type II epithelial ovarian cancers. Eur Radiol. 2022.
12. Zhu H, Ai Y, Zhang J, et al. Preoperative Nomogram for Differentiation of Histological Subtypes in Ovarian Cancer Based on Computer Tomography Radiomics. Frontiers in Oncology. 2021; 11.

13. Yu X-p, Wang L, Yu H-y, et al. MDCT-Based Radiomics Features for the Differentiation of Serous Borderline Ovarian Tumors and Serous Malignant Ovarian Tumors. Cancer Management and Research. 2021; 13:329-36.
14. Ye R, Weng S, Li Y, et al. Texture Analysis of Three-Dimensional MRI Images May Differentiate Borderline and Malignant Epithelial Ovarian Tumors. Korean Journal of Radiology. 2021; 22(1):106-17.
15. Song X-l, Ren J-L, Zhao D, Wang L, Ren H, Niu J. Radiomics derived from dynamic contrast-enhanced MRI pharmacokinetic protocol features: the value of precision diagnosis ovarian neoplasms. Eur Radiol. 2021; 31(1):368-78.
16. Park H, Qin L, Guerra P, Bay CP, Shinagare AB. Decoding incidental ovarian lesions: use of texture analysis and machine learning for characterization and detection of malignancy. Abdominal Radiology. 2021; 46(6):2376-83.
17. Li S, Liu J, Xiong Y, et al. A radiomics approach for automated diagnosis of ovarian neoplasm malignancy in computed tomography. Scientific Reports. 2021; 11(1).
18. Li NY, Shi B, Chen YL, et al. The Value of MRI Findings Combined With Texture Analysis in the Differential Diagnosis of Primary Ovarian Granulosa Cell Tumors and Ovarian Thecoma-Fibrothecoma. Front Oncol. 2021; 11:758036.
19. Jian J, Li Y, Pickhardt PJ, et al. MR image-based radiomics to differentiate type Iota and type Iota Iota epithelial ovarian cancers. Eur Radiol. 2021; 31(1):403-10.
20. Hu Y, Weng Q, Xia H, et al. A radiomic nomogram based on arterial phase of CT for differential diagnosis of ovarian cancer. Abdom Radiol (NY). 2021; 46(6):2384-92.
21. An H, Wang Y, Wong EMF, et al. CT texture analysis in histological classification of epithelial ovarian carcinoma. Eur Radiol. 2021; 31(7):5050-8.
22. Qian L, Ren J, Liu A, et al. MR imaging of epithelial ovarian cancer: a combined model to predict histologic subtypes. Eur Radiol. 2020; 30(11):5815-25.
23. Lupean R-A, Sefan P-A, Feier DS, et al. Radiomic Analysis of MRI Images is Instrumental to the Stratification of Ovarian Cysts. Journal of Personalized Medicine. 2020; 10(3).
24. Li Ya, Jian J, Pickhardt PJ, et al. MRI-Based Machine Learning for Differentiating Borderline From Malignant Epithelial Ovarian Tumors: A Multicenter Study. Journal of Magnetic Resonance Imaging. 2020; 52(3):897-904.
25. Zhang H, Mao Y, Chen X, et al. Magnetic resonance imaging radiomics in categorizing ovarian masses and predicting clinical outcome: a preliminary study. Eur Radiol. 2019; 29(7):3358-71.

26. Rundo L, Beer L, Escudero Sanchez L, et al. Clinically Interpretable Radiomics-Based Prediction of Histopathologic Response to Neoadjuvant Chemotherapy in High-Grade Serous Ovarian Carcinoma. FRONTIERS IN ONCOLOGY. 2022; 12.
27. Zargari A, Du Y, Heidari M, et al. Prediction of chemotherapy response in ovarian cancer patients using a new clustered quantitative image marker. Physics in Medicine and Biology. 2018; 63(15).
28. Danala G, Thai T, Gunderson CC, et al. Applying Quantitative CT Image Feature Analysis to Predict Response of Ovarian Cancer Patients to Chemotherapy. Academic Radiology. 2017; 24(10):1233-9.
29. Qiu Y, Tan M, McMeekin S, et al. Early prediction of clinical benefit of treating ovarian cancer using quantitative CT image feature analysis. Acta Radiologica. 2016; 57(9):1149-55.
30. Wan S, Zhou T, Che R, et al. CT-based machine learning radiomics predicts CCR5 expression level and survival in ovarian cancer. J Ovarian Res. 2023; 16(1):1.
31. Wu Y, Jiang W, Fu L, Ren M, Ai H, Wang X. Intra- and peritumoral radiomics for predicting early recurrence in patients with high-grade serous ovarian cancer. Abdom Radiol (NY). 2022.
32. Wang T, Wang H, Wang Y, et al. MR-based radiomics-clinical nomogram in epithelial ovarian tumor prognosis prediction: tumor body texture analysis across various acquisition protocols. Journal of Ovarian Research. 2022; 15(1).
33. Lu J, Cai S, Wang F, et al. Development of a prediction model for gross residual in high-grade serous ovarian cancer by combining preoperative assessments of abdominal and pelvic metastases and multiparametric MRI. Acad Radiol. 2022.
34. Li C, Wang H, Chen Y, et al. A Nomogram Combining MRI Multisequence Radiomics and Clinical Factors for Predicting Recurrence of High-Grade Serous Ovarian Carcinoma. J Oncol. 2022; 2022:1716268.
35. Hu J, Wang Z, Zuo R, et al. Development of survival predictors for high-grade serous ovarian cancer based on stable radiomic features from computed tomography images. iScience. 2022; 25(7):104628.
36. Hong Y, Liu Z, Lin D, et al. Development of a radiomic-clinical nomogram for prediction of survival in patients with serous ovarian cancer. Clin Radiol. 2022; 77(5):352-9.
37. Gao L, Jiang W, Yue Q, et al. Radiomic model to predict the expression of PD-1 and overall survival of patients with ovarian cancer. Int Immunopharmacol. 2022; 113(Pt A):109335.
38. Fotopoulou C, Rockall A, Lu H, et al. Validation analysis of the novel imaging-based prognostic radiomic signature in patients undergoing primary surgery for advanced high-grade serous ovarian cancer (HGSOC). British Journal of Cancer. 2022; 126(7):1047-54.

39. Feng S, Xia T, Ge Y, et al. Computed Tomography Imaging-Based Radiogenomics Analysis Reveals Hypoxia Patterns and Immunological Characteristics in Ovarian Cancer. Front Immunol. 2022; 13:868067.
40. Boehm KM, Aherne EA, Ellenson L, et al. Multimodal data integration using machine learning improves risk stratification of high-grade serous ovarian cancer. Nat Cancer. 2022; 3(6):723-33.
41. Avesani G, Tran HE, Cammarata G, et al. CT-Based Radiomics and Deep Learning for BRCA Mutation and Progression-Free Survival Prediction in Ovarian Cancer Using a Multicentric Dataset. Cancers (Basel). 2022; 14(11).
42. Yu XY, Ren J, Jia Y, et al. Multiparameter MRI Radiomics Model Predicts Preoperative Peritoneal Carcinomatosis in Ovarian Cancer. Frontiers in Oncology. 2021; 11.
43. Yi X, Liu Y, Zhou B, et al. Incorporating SULF1 polymorphisms in a pretreatment CT-based radiomic model for predicting platinum resistance in ovarian cancer treatment. Biomedicine & Pharmacotherapy. 2021; 133.
44. Song X-L, Ren J-L, Yao T-Y, Zhao D, Niu J. Radiomics based on multisequence magnetic resonance imaging for the preoperative prediction of peritoneal metastasis in ovarian cancer. Eur Radiol. 2021; 31(11):8438-46.
45. Liu M, Ge Y, Li M, Wei W. Prediction of BRCA gene mutation status in epithelial ovarian cancer by radiomics models based on 2D and 3D CT images. Bmc Medical Imaging. 2021; 21(1).
46. Li MR, Liu MZ, Ge YQ, Zhou Y, Wei W. Assistance by Routine CT Features Combined With 3D Texture Analysis in the Diagnosis of BRCA Gene Mutation Status in Advanced Epithelial Ovarian Cancer. Frontiers in Oncology. 2021; 11.
47. Li HM, Gong J, Li RM, et al. Development of MRI-Based Radiomics Model to Predict the Risk of Recurrence in Patients With Advanced High-Grade Serous Ovarian Carcinoma. American Journal of Roentgenology. 2021; 217(3):664-75.
48. Li H, Zhang R, Li R, et al. Noninvasive prediction of residual disease for advanced high-grade serous ovarian carcinoma by MRI-based radiomic-clinical nomogram. Eur Radiol. 2021; 31(10):7855-64.
49. Chen H-z, Wang X-r, Zhao F-m, et al. A CT-based radiomics nomogram for predicting early recurrence in patients with high-grade serous ovarian cancer. European Journal of Radiology. 2021; 145.
50. Chen H-z, Wang X-r, Zhao F-m, et al. The Development and Validation of a CT-Based Radiomics Nomogram to Preoperatively Predict Lymph Node Metastasis in High-Grade Serous Ovarian Cancer. Frontiers in Oncology. 2021; 11.
51. Ai Y, Zhang J, Jin J, Zhang J, Zhu H, Jin X. Preoperative Prediction of Metastasis for Ovarian Cancer Based on Computed Tomography Radiomics Features and Clinical Factors. Frontiers in Oncology. 2021; 11.

52. Veeraraghavan H, Vargas HA, Alejandro-Jimenez S, et al. Integrated Multi-Tumor Radio-Genomic Marker of Outcomes in Patients with High Serous Ovarian Carcinoma. Cancers. 2020; 12(11).
53. Wei W, Liu Z, Rong Y, et al. A Computed Tomography-Based Radiomic Prognostic Marker of Advanced High-Grade Serous Ovarian Cancer Recurrence: A Multicenter Study. Frontiers in Oncology. 2019; 9.
54. Meier A, Veeraraghavan H, Nougaret S, et al. Association between CT-texture-derived tumor heterogeneity, outcomes, and BRCA mutation status in patients with high-grade serous ovarian cancer. Abdominal Radiology. 2019; 44(6):2040-7.
55. Lu H, Arshad M, Thornton A, et al. A mathematical-descriptor of tumor-mesoscopic-structure from computed-tomography images annotates prognostic- and molecular-phenotypes of epithelial ovarian cancer. Nature Communications. 2019; 10.
56. Rizzo S, Botta F, Raimondi S, et al. Radiomics of high-grade serous ovarian cancer: association between quantitative CT features, residual tumour and disease progression within 12 months. Eur Radiol. 2018; 28(11):4849-59.
57. Vargas HA, Veeraraghavan H, Micco M, et al. A novel representation of inter-site tumour heterogeneity from pre-treatment computed tomography textures classifies ovarian cancers by clinical outcome. Eur Radiol. 2017; 27(9):3991-4001.
